# Screening and Analysis of Janelia FlyLight Project Enhancer-Gal4 Strains Identifies Multiple Gene Enhancers Active During Hematopoiesis in Normal and Wasp-Challenged *Drosophila* Larvae

**DOI:** 10.1534/g3.116.034439

**Published:** 2016-12-01

**Authors:** Tsuyoshi Tokusumi, Yumiko Tokusumi, Mark S. Brahier, Victoria Lam, Jessica R. Stoller-Conrad, Paul T. Kroeger, Robert A. Schulz

**Affiliations:** Department of Biological Sciences, University of Notre Dame, Indiana 46556

**Keywords:** blood cell-specific gene expression, *Drosophila* hematopoiesis, gene enhancer-Gal4 line screening, larval hemocyte essential genes, wasp-challenged larvae

## Abstract

A GFP expression screen has been conducted on >1000 Janelia FlyLight Project enhancer-Gal4 lines to identify transcriptional enhancers active in the larval hematopoietic system. A total of 190 enhancers associated with 87 distinct genes showed activity in cells of the third instar larval lymph gland and hemolymph. That is, gene enhancers were active in cells of the lymph gland posterior signaling center (PSC), medullary zone (MZ), and/or cortical zone (CZ), while certain of the transcriptional control regions were active in circulating hemocytes. Phenotypic analyses were undertaken on 81 of these hematopoietic-expressed genes, with nine genes characterized in detail as to gain- and loss-of-function phenotypes in larval hematopoietic tissues and blood cells. These studies demonstrated the functional requirement of the *cut* gene for proper PSC niche formation, the *hairy*, *Btk29A*, and *E2F1* genes for blood cell progenitor production in the MZ domain, and the *longitudinals lacking*, *dFOXO*, *kayak*, *cap-n-collar*, and *delilah* genes for lamellocyte induction and/or differentiation in response to parasitic wasp challenge and infestation of larvae. Together, these findings contribute substantial information to our knowledge of genes expressed during the larval stage of *Drosophila* hematopoiesis and newly identify multiple genes required for this developmental process.

The UAS-Gal4 system is an excellent research tool to analyze gene expression and function in *Drosophila* (Brand and Perrimon 1993). Recently, large-scale collections of Gal4 strains, such as Janelia FlyLight and Vienna Tile Gal4 lines, have been established expanding the breadth of these analyses (Jenett *et al.* 2012; Jory *et al.* 2012; Kvon *et al.* 2014; Manning *et al.* 2012; Pfeiffer *et al.* 2008). Unlike previous enhancer trap strains, these newer transgenic lines have relatively small DNA fragments (∼2–3 kb) linked to Gal4 genes. This approach has several advantages, allowing researchers to view gene expression patterns in defined tissues, identify regulatory regions to direct gene expression in specific cells, and use tissue-specific tools, such as a Gal4 driver, to induce the expression of interesting genes in target tissues. In this study, we performed an enhancer-Gal4 strain screen with a focus on select hematopoietic tissues, those being the lymph glands and hemolymph of third instar larvae.

During *Drosophila* embryonic development, the cephalic mesoderm gives rise to hemocytes and these blood cells are contributed to the hemolymph of larval stage animals. The lymph gland is the larval hematopoietic organ, being composed of multiple paired lobes. In third instar larvae, the primary lobes of the lymph gland consist of three parts (Figure 1, A and B): the CZ, the MZ, and the PSC (Jung *et al.* 2005). The CZ is occupied by mature blood cells, while the MZ is composed of a heterogeneous population of blood progenitor cells (Krzemien *et al.* 2010; Tokusumi *et al.* 2011; Benmimoun *et al.* 2015; Oyallon *et al.* 2016). In contrast, the PSC functions as a hematopoietic stem cell-like niche for the hematopoietic progenitors. To maintain blood progenitor cells, the JAK/STAT, Hedgehog (Hh), Insulin-like receptor (InR), Wingless (Wg), Pvf/Pvr, and fibroblast growth factor (FGF) pathways and ROS signaling are key regulators (Benmimoun *et al.* 2012; Dragojlovic-Munther and Martinez-Agosto 2012, 2013; Krzemień *et al.* 2007; Mandal *et al.* 2007; Mondal *et al.* 2011, 2014; Owusu-Ansah and Banerjee 2009; Shim *et al.* 2012; Sinenko *et al.* 2009). In addition, our previous work has shown that the germ line differentiation factor bag-of-marbles (*bam*) and microRNA-7 (*mir-7*) cooperatively regulate blood progenitor cells and their differentiation (Tokusumi *et al.* 2011). In the PSC, two transcription factors, Antennapedia (Antp) and Knot/Collier (Col), play important roles in PSC development and maintenance (Krzemień *et al.* 2007; Mandal *et al.* 2007). Col likewise functions in a cell-autonomous manner to maintain the hematopoietic progenitor population (Benmimoun *et al.* 2015). The Decapentaplegic (Dpp), InR, Wg, and Slit/Robo signaling pathways are also key regulators of PSC size and organization (Benmimoun *et al.* 2012; Morin-Poulard *et al.* 2016; Pennetier *et al.* 2012; Sinenko *et al.* 2009; Tokusumi *et al.* 2012, 2015).

**Figure 1:**
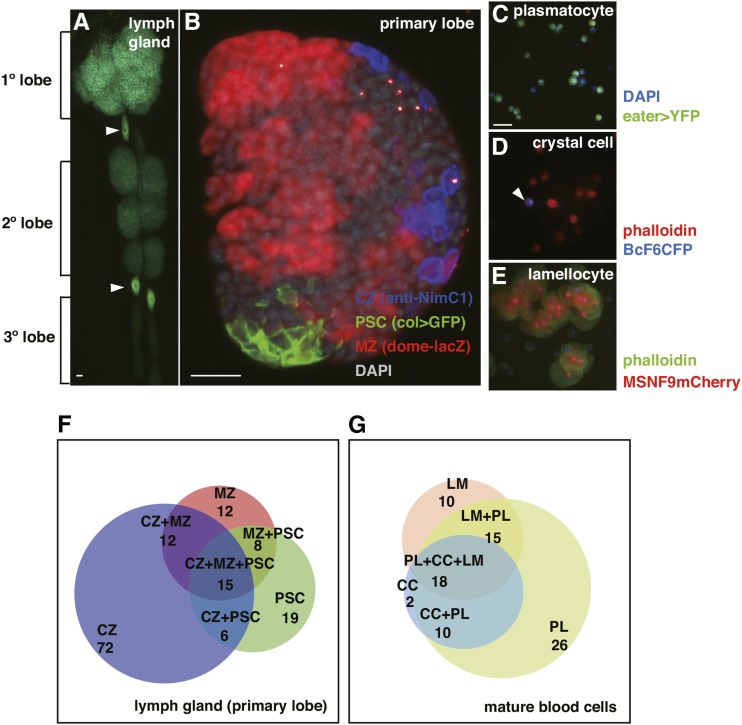
Lymph gland structure, cellular domains, blood cell types, and the results of the enhancer-Gal4 line screen. (A) Organization of the lymph glands into primary, secondary, and tertiary lobes. The primary lobe is positive for the plasmatocyte marker *eater-GFP*, while the arrowheads indicate positive pericardial cells as well. (B) Cellular and functional domains within the primary lobe of a midthird instar larval lymph gland. The primary lobe is composed of three parts: the CZ (blue) marked by NimC1 protein expression, MZ (red) marked by domeMESO expression, and the PSC (green) marked by *col > GFP* expression. Gray corresponds to DAPI labeling. (C–E) Three mature hemocyte types are found in the CZ and hemolymph. (C) Plasmatocytes (green) are marked by *eater > YFP* expression. Blue corresponds to DAPI labeling. (D) Crystal cells (blue) are marked by BcF6CFP expression. Red corresponds to phalloidin detection of plasmatocytes. (E) Lamellocytes (red) are labeled by MSNF9mCherry expression. Green indicates phalloidin staining of these large cells. (F and G) Summary of the enhancer-Gal4 > UAS-GFP-expressing lines as to (F) expression in cells of the primary lymph gland lobe and (G) blood cells found in the circulating hemolyph under normal or wasp-challenged growth conditions. Bar, 20 μm in all images. CZ, cortical zone; DAPI, 4’,6-diamidino-2-phenylindole; GFP, green fluorescent protein; MZ, medullary zone; PSC, posterior signaling center; YFP, yellow fluorescent protein.

Differentiation of hematopoietic progenitors can generate three mature blood cell types in *Drosophila* (Figure 1, C–E): plasmatocytes, crystal cells, and lamellocytes (Evans *et al.* 2003). Plasmatocytes are small round cells with phagocytic capacity and they constitute the majority of circulating hemocytes. Crystal cells carry prophenol oxidase, which is involved in melanization. Lamellocytes are large flat adherent cells that are rare under normal developmental and physiological conditions. However, under challenge conditions such as wasp parasitization, numerous lamellocytes are induced, wherein they function to encapsulate the foreign invader. In this study, we crossed > 1000 Janelia FlyLight Project enhancer-Gal4 lines with the UAS-GFP reporter line and documented GFP expression patterns in both lymph glands and larval hemolymph. Enhancer activity was classified as to the positive hemocyte expression type (plasmatocyte, crystal cell, and/or lamellocyte) and distinct cell-specific expression in domains of the lymph glands (CZ, MZ, PSC, and/or posterior lobes). These studies provided a wealth of information on gene enhancers that are active in cells of the larval hematopoietic system, and multiple associated genes were further studied and shown to be essential for proper hematopoiesis in normal or wasp-challenged larvae.

## Materials and Methods

### Fly and wasp lines

Enhancer-Gal4 lines of the Janelia FlyLight Project (http://flweb.janelia.org/), *UAS*-RNAi TRiP lines, *UAS-GFP*, *UAS-mCD8*::*GFP*, *Pxn-Gal4*, *UAS-E2f1 UAS-Dp*, and *UAS-E2f1^PIP-3A^* were obtained from the Bloomington *Drosophila* Stock Center. The *TepIV-Gal4* line was obtained from the DGRC (Kyoto, Japan). We also used the following fly strains: domeMESO (dome-lacZ) (Hombria *et al.* 2005); *eater-GFP* (Sorrentino *et al.* 2007); *MSNF9-mCherry* (Tokusumi *et al.* 2009a); *Pcol85* (Krzemień *et al.* 2007); *UAS-FOXO*, *dFOXO^21^*, and *dFOXO^25^* (Junger *et al.* 2003); *UAS-cncB* (Veraksa *et al.* 2000); *UAS-cncC* (Sykiotis and Bohmann 2008); and *puc^E69^ kay^1^* and *kay^2^* (Zeitlinger and Bohmann 1999). To generate BcF6-mCherry lines, we subcloned the BcF6 DNA regulatory region into pmCherry Pelican, injected *w^1118^* embryos, and generated transgenic lines (Gajewski *et al.* 2007; Tokusumi *et al.* 2009a). Hymenoptera wasp *L. boulardi*, strain Lb17, was provided by T. A. Schlenke and S. Govind (Schlenke *et al.* 2007).

### Tissue immunostaining

Lymph gland immunostaining was performed as previously described (Tokusumi *et al.* 2011). The following primary antibodies were used: mouse anti-Antp (1:100; 4C3, Developmental Studies Hybridoma Bank), anti-β-Galactosidase (1:100; Promega), and mouse anti-NimC1 antibody (Vilmos *et al.* 2004) (1:100; I. Ando). As secondary antibodies, we used the Alexa 555-conjugated anti-mouse IgG antibody (Invitrogen). We analyzed stained samples with a Zeiss Axioplan Fluorescence microscope or a Nikon AR-1 laser-scanning confocal microscope. Data were collected from at least 10 third instar larvae in all enhancer expression or gene phenotype analyses experiments.

### Wasp infestation

Parasitoid wasp *Leptopilina boulardi* strain Lb17 was reared in *Drosophila w^1118^* flies (Schlenke *et al.* 2007). Infestation experiments were performed as described previously (Sorrentino *et al.* 2002). Briefly, 36–48 hr old larvae were exposed to 8–10 female wasps for 24 hr at 25°, left for 2 d at room temperature, and dissected. As shown in Figure 7, lymph glands were categorized by three levels of MSNF9mCherry-positive cells as it was difficult to count lamellocytes precisely due to their aggregation.

### Data availability

FlyLight Project GAL4 lines are available from the Bloomington *Drosophila* Stock Center. Several lines used in this study are maintained in our lab and available upon request. Both *BcF6mCherry* and *MSNF9mCherry* fly lines are available upon request. 

## Results and Discussion

### Enhancer-Gal4 line screening strategy

The Janelia FlyLight Project has generated transgenic Gal4 lines containing ∼7000 enhancers from ∼1200 genes (Jenett *et al.* 2012; Pfeiffer *et al.* 2008). Previously, we performed a microarray analysis of RNAs present in lymph glands and confirmed at least 8000 genes being active in this larval hematopoietic organ (Tokusumi *et al.* 2011). Approximately 400 lymph gland-expressed genes overlap with those analyzed in the FlyLight Project and there are ∼3000 enhancer-Gal4 lines related to these genes. We chose to study transcription factor and signaling pathway genes, selected 206 genes/1096 enhancer-Gal4 lines for analysis, and crossed these strains with *UAS-GFP* flies to monitor resulting Gal4 activity patterns in at least 10 larvae (Supplemental Material, Table S1). We identified 190 enhancers that can direct GFP reporter gene expression in larval blood tissues, including lymph glands and hemolymph. These findings are summarized in Figure 1 and Table S2. Among the larval blood cell-positive enhancer-Gal4 strains, 144 lines were expressed in cells present in the primary lobes of the lymph glands, whereas 81 lines showed enhancer-Gal4 activity in cells of the hemolymph and/or sessile hemocytes (Figure 1, F and G).

#### PSC-expressed enhancer-Gal4 lines:

**Figure 2:**
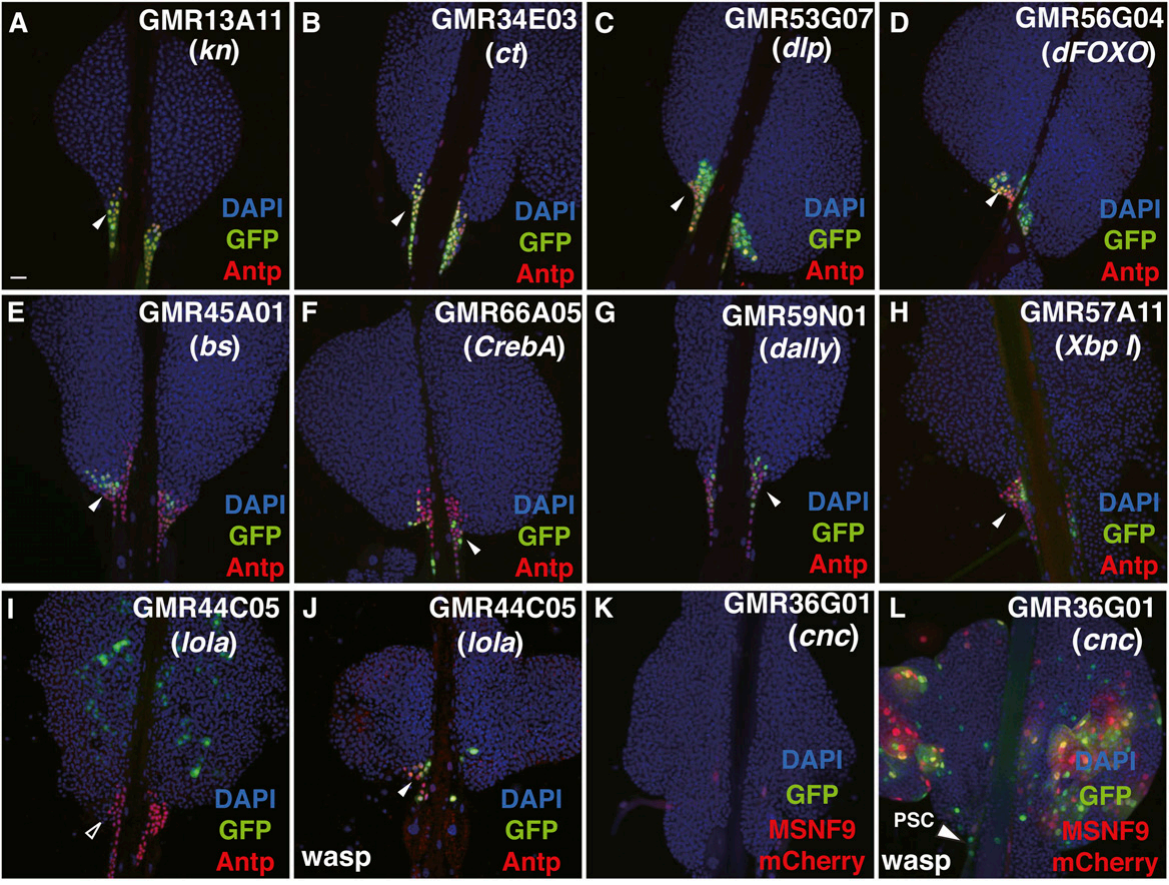
PSC-expressed enhancer-Gal4 lines. (A–L) The various lines tested are indicated in the panels with the enhancer location as to genetic locus noted below the strain name. (A–D) Lines expressing the GFP marker (green) with a strong or complete presence in cells expressing the Antp PSC protein marker (red). (E–H) Lines expressing the GFP reporter in a subset of Antp^+^ PSC cells. (I–L) Wasp infestation can induce enhancer activity and reporter gene expression in PSC cells. (I) Under normal growth conditions, the GMR44C05 line shows GFP expression in CZ cells but not PSC cells. (J) Post-wasp infestation, the *lola* gene enhancer is active in PSC cells. (K) The GMR36G01 line fails to show any expression in cells of the lymph gland. (L) Post-wasp challenge, the *cnc* enhancer is active in both PSC cells and lamellocytes, the latter marked by the MSNF9mCherry transgene (red). Arrowheads indicate PSC cells expressing GFP and/or Antp. Bar, 20 μm in all images. Antp, Antennapedia; CZ, cortical zone; DAPI, 4’,6-diamidino-2-phenylindole; GFP, green fluorescent protein; PSC, posterior signaling center.

We identified 48 lines with enhancer-Gal4 activity in the PSC domain of the lymph gland. Of these, 19 enhancers induced Gal4 expression solely in PSC cells, whereas 29 other enhancer-Gal4 combinations were expressed in both the PSC and other cells present within the primary lymph gland lobes (Figure 1F, Figure S1, and Table S2). For examples from the former group, enhancer-Gal4 expression in lymph glands from the GMR13A11, GMR34E03, and GMR53G07 strains (Figure 2, A–C) was highly consistent with Antp-positive PSC cells, while enhancer-Gal4 activity in tissues from the GMR56G04, GMR45A01, GMR66A05, GMR59N01, and GMR57A11 lines (Figure 2, D–H) overlapped with a subset of Antp-positive PSC cells. The efficacy of this screen could be immediately assessed as the GMR13A11 strain showed the expression of a *kn/col* gene enhancer in PSC cells, with the Col transcriptional regulator known to function in PSC and hematopoietic progenitor population maintenance (Benmimoun *et al.* 2015: Krzemień *et al.* 2007). Likewise, the GMR53G07 and GMR59N01 strains show the activity of *dally-like (dlp)* and *dally* gene enhancers in niche cells, consistent with the function of these Dpp signaling pathway genes in PSC size control and organization (Morin-Poulard *et al.* 2016; Pennetier *et al.* 2012). The implication is that other PSC-expressed enhancers are likely associated with genes that function in some manner in cells of the hematopoietic niche.

In addition, we identified three enhancer regions that directed Gal4 activity in PSC cells post-wasp egg infestation, but not under normal developmental conditions. Specifically, the GMR44C05 and GMR44B09 strains associated with the *longitudinals lacking* (*lola*) gene possessed enhancers active in CZ cells in control animals (Figure 2I), but wasp parasitization resulted in induced GFP expression in PSC cells while also causing a reduction in CZ cell number (Figure 2J and Figure S1). Comparably, the GMR36G01 strain contains an enhancer associated with the *cap-n-collar* (*cnc*) gene. This enhancer is inactive in cells of the lymph gland of wild-type third instar larvae (Figure 2K), but the enhancer activates GFP expression in PSC cells and lamellocytes post-wasp challenge (Figure 2L). Thus, the *cnc* gene may be a locus that is induced in niche cells and defensive lamellocytes upon wasp infestation, warranting an analysis of its function in the hematopoietic system under this means of physiological stress to third instar larvae.

#### MZ-expressed enhancer-Gal4 lines:

**Figure 3:**
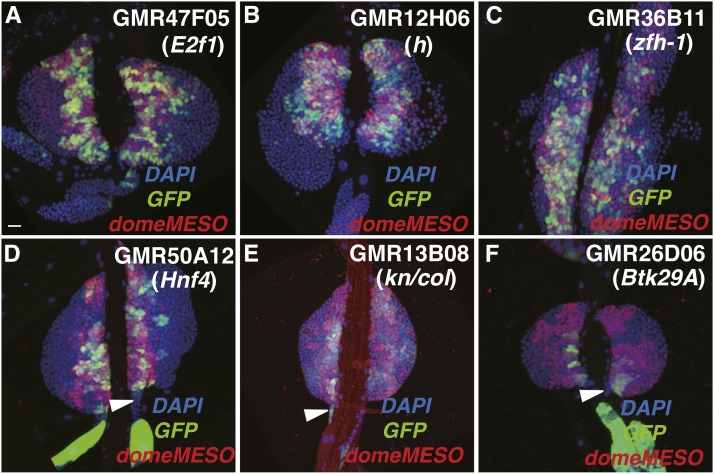
MZ-expressed enhancer-Gal4 lines. (A–F) The various lines tested are indicated in the panels with the enhancer location as to genetic locus noted below the strain name. GFP expression patterns (green) of (A) GMR47F05, (B) GMR12H06, (C) GMR36B11, and (D) GMR50A12 are consistent with enhancer activity in all or most domeMESO^+^ MZ cells (red). The (E) GMR13B08 and (F) GMR26D06 lines show GFP expression in a subset of domeMESO^+^ cells. Bar, 20 μm in all images. DAPI, 4’,6-diamidino-2-phenylindole; GFP, green fluorescent protein; MZ, medullary zone.

We identified 47 Gal4 lines that showed GFP reporter gene expression in the MZ domain (Figure 1F). Among these lines, 12 enhancer regions induced the reporter gene exclusively in MZ cells (Figure S1 and Table S2). We observed that enhancers present in the GMR47F05, GMR12H06, GMR36B11, and GMR50A12 lines drove the GFP reporter strongly in MZ cells marked by domeMESO expression (Figure 3, A–D). These MZ-active enhancers are associated with genes encoding the E2f1, Hairy (H), Zfh-1, and Hnf4 transcriptional regulators, with functional studies documenting an importance for these protein factors in MZ cell production or maintenance (discussed below). The GMR13B08 strain, which contains an enhancer DNA located in an intron of the *col* gene, showed weak GFP expression in cells of both the MZ and PSC domains (Figure 3E). Again, pertaining to the efficacy of the current screen, a recent report demonstrated that the Col transcriptional regulator is weakly expressed in MZ cells, where it is known to function in the maintenance of blood progenitor cells (Benmimoun *et al.* 2015). Thus, this may be the genomic DNA region that is responsible for Col expression in this lymph gland domain.

It should be noted that the MZ is composed of both core blood cell progenitors (Benmimoun *et al.* 2015; Oyallon *et al.* 2016) and more peripheral intermediary progenitors (Krzemien *et al.* 2010; Tokusumi *et al.* 2011). In testing for enhancers active in this lymph gland domain, we utilized the commonly used domeMESO (dome-lacZ) marker, which is expressed in the majority of MZ cells but not in all Yan-positive intermediary cells. Thus, our findings do not readily discriminate between enhancers that are active in core progenitors and/or intermediary progenitors.

#### CZ and hemolymph blood cell-expressed enhancer-Gal4 lines:

**Figure 4:**
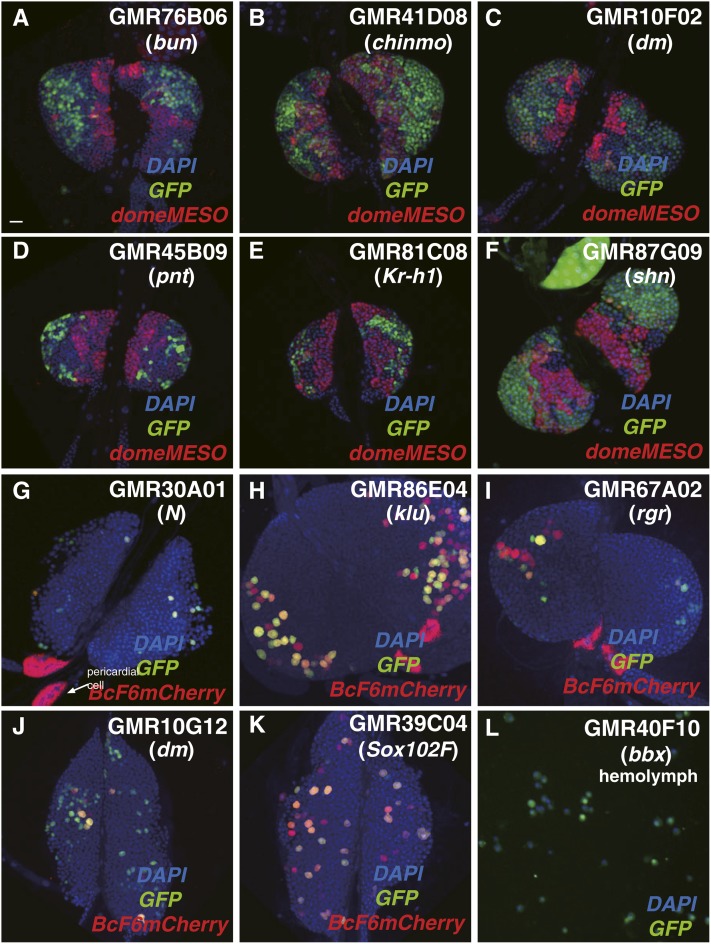
CZ and specific blood cell type-expressed enhancer-Gal4 lines. (A–L) The various lines tested are indicated in the panels with the enhancer location as to genetic locus noted below the strain name. (A–F) Representative lines that express the GFP reporter (green) in peripheral CZ cells that do not overlap with domeMESO + MZ cells (red). Lymph glands are also stained with the nuclear marker DAPI (blue). (G–K) Five enhancer-Gal4 lines showing GFP expression in mature crystal cells, marked by the activity of the crystal cell-specific transgene BcF6-mCherry (red). (L) The GMR40F10 line, harboring an enhancer from the *bbx* gene, fails to show expression in CZ cells of the lymph gland but is solely expressed in circulating plasmatocytes. Bar, 20 μm in all images. CZ, cortical zone; DAPI, 4’,6-diamidino-2-phenylindole; GFP, green fluorescent protein; MZ, medullary zone.

We identified 105 lines with enhancer-Gal4 activity in the CZ domain of the primary lymph gland, which is composed of differentiating or mature blood cells (Figure 1F). Among these, 72 lines showed GFP expression exclusively in CZ cells. We observed that enhancers present in the GMR76B06, GMR41D08, GMR10F02, GMR45B09, GMR81C08, and GMR87G09 strains drove the GFP reporter strongly in CZ cells, peripheral to MZ cells marked by domeMESO expression (Figure 4, A–F). These six enhancer DNAs are associated with the *bun*, *chinmo*, *dm*, *pnt*, *Kr-h1*, and *shn* genes, respectively, all of which encode transcription factors of different structural domain classes.

Colabeling of GFP with the crystal cell marker *BcF6mCherry* allowed for the identification of enhancers that were active in differentiating or mature crystal cells. The GMR30A01 enhancer corresponds to a genomic DNA located in an intron of the *Notch* gene. GFP expression in lymph glands from this line is expressed in *BcF6-mCherry*-labeled crystal cells (Figure 4G), but also a few plasmatocytes marked by an anti-NimC1 antibody (Table S2). GMR86E04 contains an enhancer derived from the *klumpfuss* (*klu*) gene, GMR67A02 contains an enhancer of the *regular* (*rgr*) gene, and GMR10G12 an enhancer from the *Myc/dm* gene. All three genes encode transcriptional regulators and the corresponding lines showed clear GFP expression in crystal cells (Figure 4, H–J) and also in circulating plasmatocytes (Table S2). It is interesting that the *Notch* and *Myc/dm* enhancers are active in crystal cells as the latter gene contains genomic DNA regions that contain Notch-response elements that are bound by Su(H) transcriptional regulator (Terriente-Felix *et al.* 2013). In addition, we found that the GMR39C04 strain, which contains an enhancer DNA associated with the *Sox102F* gene, directed GFP expression in crystal cells (Figure 4K). This line also showed GFP expression in circulating plasmatocytes (Table S2).

A total of 81 lines showed GFP expression in blood cells of the hemolymph of third instar larvae (Figure 1G), and most showed GFP reporter expression in both lymph gland CZ cells and hemolymph blood cells. One interesting strain was GMR40F10, containing an enhancer associated with the *bobby sox* (*bbx*) gene, which encodes an HMG box class transcription factor. GFP expression was observed in circulating plasmatocytes (Figure 4L), but not in any cells of the lymph glands (Table S2), making this a unique strain and enhancer discovered in this screen.

**Figure 5:**
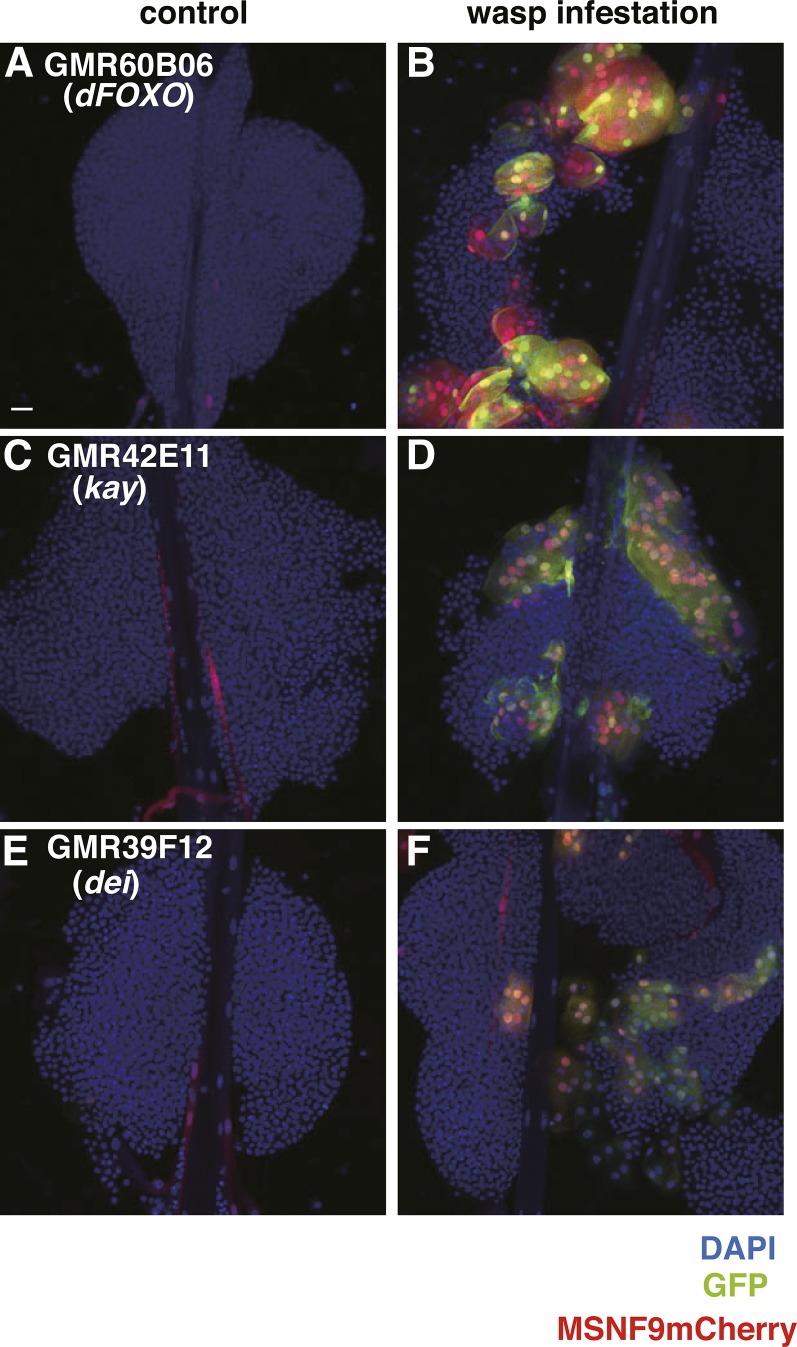
Lamellocyte-expressed enhancer-Gal4 lines. (A, C, and E). Various lines tested are indicated in the panels with the enhancer location as to genetic locus noted below the strain name. Three lines are presented that failed to show GFP expression (green) in any cell type of the lymph gland. (B, D, and F). Post-wasp infestation, lamellocytes identified by MSNF9mCherry transgene expression (red) are induced in high numbers. Lymph glands are also stained with the nuclear marker DAPI (blue). Bar, 20 μm in all images. DAPI, 4’,6-diamidino-2-phenylindole; GFP, green fluorescent protein.

Finally, we identified 43 lines that showed GFP expression in lamellocytes induced upon wasp infestation and 11 that showed exclusive expression only in this blood cell type. Three examples are given in Figure 5, wherein the enhancer-Gal4 combinations were inactive in control lymph glands but induced to high activity levels in lamellocytes post-wasp infestation. These included GMR60B06 (enhancer from the *dFOXO* gene), GMR42E11 [enhancer from the *kayak* (*kay*) gene], and GMR39F12 [enhancer from the *delilah* (*dei*) gene] (Figure 5, A–F). These three genes encode transcription factors and the specific *de novo* induction that results suggests a role for one or more of these regulators in the production of the defensive lamellocyte population in response to parasitic wasp challenge to larvae.

### Functional analyses of enhancer-associated genes

We identified 190 enhancers that showed activity in cells of the larval hematopoietic system, with these transcriptional regulatory regions associated with 87 distinct genes. The hematopoietic functions of some of these genes have been previously documented, but the functions of many of the blood cell-active genes identified in this screen have not been described. Therefore, we undertook phenotypic analyses of 81 of these genes using gain- and loss-of-function approaches possible with the Gal4-UAS gene expression system (summarized in Table 1, Table 2, Table 3, and Table S3). We collected data from at least 10 larvae of each genotype. The hematopoietic functions of nine of these genes are presented in more detail as follows.

**Table 1:** Summary of transcriptional enhancers active in PSC cells and phenotype analyses of their associated genes in the PSC lymph gland domain

Gene	Mutant	RNAi	GOF	Gene	Mutant	RNAi	GOF
*Alh*		NE		*kay*		△	
*br*		▽		*kn/col*		NE	
*bs*		NE		*LanA*		NE	
*BtbVII*		NE		*lola*		▼	LM
*Btk29A*		△		*Mad*		▼	
*crebA*		NE	▼	*Max*		NE	
*CrebB17A*		△	▼	*pnt*		△	
*cnc*		NE	▲*^a^*, ▼*^b^*	*ptc*		△	
*ct*		△	▼	*rgr*		NE	
*cwo*		NE		*sbb*		NE	
*Dad*		NE		*STAT92E*		△	
*dally*		NE		*Stj*		△	
*dlp*		NE		*Trl*		△	
*Dp*	▼	▼	▲*^c^*	*ttk*		▽*	
*E2f*	▼	▼	▲*^c^*,*^d^*	*Vri*		NE	
*EcR*		NE		*Xbp1*		▼	
*Eip75B*		▲		*Xrp1*		▽	
*FoxO*	▲*^e^*	NE		*Zfh1*		NE	
*Hnf4*		NE					

RNAi, RNA interference; GOF, gain-of-function; NE, no effect; △, bigger PSC cell; ▽, minor decrease of PSC; ▼, strong decrease of PSC; LM, lamellocyte production; ▲, strong increase of PSC; ▽*, weak *hhGFP* expression.

a *UAS-cncB*.

b *UAS-cncC*.

c *UAS-E2f1 UAS-Dp*.

d *UAS-E2f1^PIP-3A^*.

e *dFOXO^21^/dFOXO^25^* (Tokusumi *et al.* 2012) and strong *hhGFP* expression.

**Table 2:** Summary of transcriptional enhancers active in MZ cells and phenotype analyses of their associated genes in the MZ lymph gland domain

Gene	Mutant	RNAi	GOF	Gene	Mutant	RNAi	GOF
*Alh*		▼		*H*		▼	
*apt*		NE		*Hnf4*		▽	
*br*		▽		*hth*		▽	
*BtbVII*		▼		*Jumu*		▼	
*Btk29A*		▼		*kay*		▽	
*CG10200*		NE		*kn/col*		NE	
*chinmo*		▼		*LanA*		▼	
*cnc*		▼		*Mad*		NE	
*ct*		NE		*pnt*		▽	
*cwo*		▼		*sbb*		NE	
*Dp*	▼	▽	▲*^a^*, LM*^a^*	*Smox*		NE	
*E2f1*	▼, small LG	▼	▲*^a^*, LM*^a^*	*Trl*		▼	
*EcR*		NE		*ttk*		▼	
*Eip75B*		NE		*unc-5*		NE	
*ems*		NE		*Vri*		NE	
*FoxO*		NE		*Xrp1*		▼	
*gish*		▼		*Zfh1*		▽	

RNAi, RNA interference; GOF, gain-of-function; ▼, strong decrease of MZ; NE, no effect; ▽, minor decrease of MZ; ▲, strong increase of MZ; LM, lamellocyte production; LG, lymph gland.

a *UAS-E2f1 UAS-Dp*.

b *UAS-E2f1^PIP-3A^*.

**Table 3:** Summary of transcriptional enhancers active in CZ cells, hemolymph blood cells, or lamellocytes, and phenotype analyses of their associated genes

Gene	Mutant	RNAi	GOF	Gene	Mutant	RNAi	GOF	Gene	Mutant	RNAi	GOF
*Alh*		NE		*Dp*		NE		*mts*		LM	
*bbx*		NE		*E2f1*	LM	LM		*N*		NE	
*bon*		NE		*EcR*		LM		*osa*		LM	
*br*		LM		*Eip75B*		LM ▼		*pnt*		NE	
*bs*		NE		*dFOXO*	LM ▼	LM ▼	LM	*rgr*		NE	
*BtbVII*		NE		*gish*		LM		*rho*		NE	
*bun*		NE		*HLH106*		NE		*sbb*		NE	
*CadN*		NE		*HLHm3*		NE		*shn*		NE	
*CG10200*		LM		*hth*		NE		*Smox*		LM	
*CG10543*		LM		*InR*		LM		*Sox102F*		NE	
*CG1129*		LM		*Jra*		NE		*Stj*		NE	
*CG32613*		NE		*Jumu*		NE		*tna*		NE	
*chinmo*		LM		*katanin60*		NE		*Trl*		NE	
*cnc*		LM ▼	LM*^a^*,*^b^*	*kay*	LM ▼	LM ▼	LM	*ttk*		NE	
*crebA*		NE		*klu*		NE		*twi*		LM	
*ct*		LM		*l(2)gl*		LM		*usp*		NE	
*CTPsym*		LM		*LanA*		NE		*usp*		NE	
*cwo*		NE		*lola*		NE		*Vap*		LM	
*d4*		NE		*lz*		NE		*Vap*		NE	
*dei*		LM ▼	LM	*Mad*		NE		*Vri*		NE	
*dlp*		NE		*mamo*		NE		*Xrp1*		NE	
*dm*		LM		*Max*		NE		*yan (aop)*		NE	
*Dscam*		LM		*Mef2*		LM		*Zfh1*		LM	

RNAi, RNA interference; GOF, gain-of-function; NE, no effect; LM, lamellocyte production; LM ▼, decrease lamellocytes under wasp infestation.

a *UAS-cncB*.

b *UAS-cncC*.

#### cut (ct):

**Figure 6:**
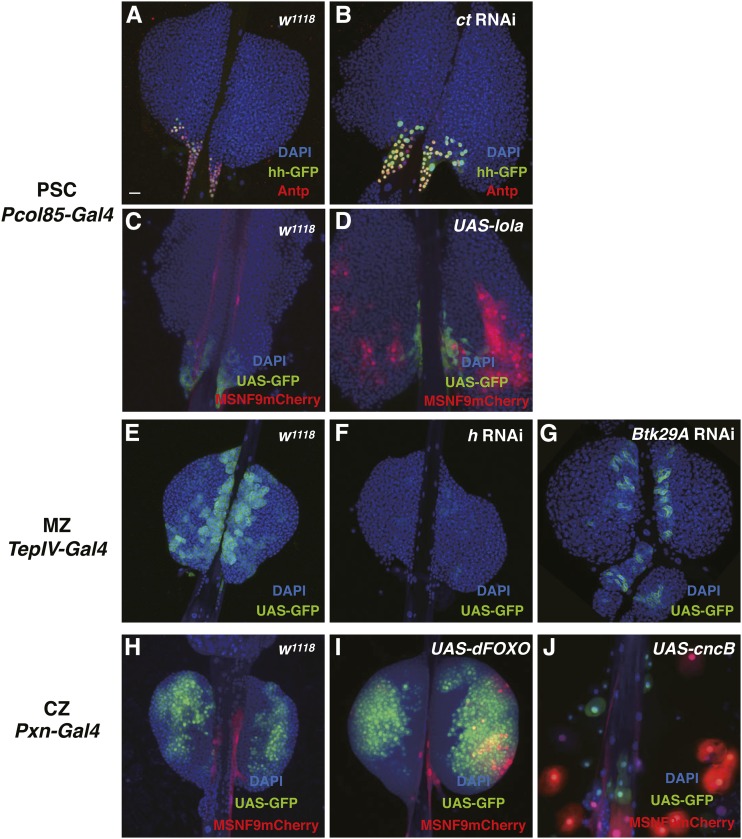
Phenotype analyses of select PSC, MZ, and lamellocyte-expressed genes. (A–D) PSC-expressed or activated genes, with lymph glands stained with nuclear DAPI (blue), PSC markers *hh-GFP* (green), Antp (red), or GFP (green), and lamellocyte marker *MSNF9mCherry* (red). (A and C) Control lymph glands. (B) *cut* knockdown lymph glands through *col-Gal4 > UAS cut* RNAi expression. (D) *lola* gain-of-function lymph glands through *col-Gal4 > UAS lola* expression. (E–G) Knockdown phenotypes of MZ-expressed genes. Lymph glands were stained for nuclear DAPI (blue) and mCD8::GFP (green). (E) Control lymph glands. (F) *h* knockdown lymph glands through *TepIV-Gal4 > UAS h* RNAi expression. (G) *Btk29A* knockdown lymph glands through *TepIV-Gal4 > UAS Btk29A* RNAi expression. (H–J) Examples of lamellocyte-inducing genes. Lymph glands were stained with nuclear DAPI (blue), GFP (green), and the lamellocyte marker *MSNF9mCherry* (red). (H) Control lymph glands. (I) *dFOXO* gain-of-function lymph glands through *Pxn-Gal4 > UAS dFOXO* expression. (J) *cnc* gain-of-function lymph glands through *Pxn-Gal4 > UAS cncB* expression. Bar, 20 μm in all images. Antp, Antennapedia; CZ, cortical zone; DAPI, 4’,6-diamidino-2-phenylindole; GFP, green fluorescent protein; MZ, medullary zone; PSC, posterior signaling center; RNAi, RNA interference.

The GMR34E03 line showed strong GFP reporter expression in PSC cells (Figure 2B). This line contains an enhancer corresponding to *ct* gene DNA. PSC-specific knockdown of *ct* function by *col-Gal4 > UAS ct* RNAi expression resulted in a strong overproduction of PSC cells (Figure 6B). Conversely, gain-of-function *ct* due to *col-Gal4 > UAS ct* expression led to a decrease in PSC cell number (Table 1). Thus, the homeobox protein Ct appears to play a negative role in the production and/or proliferation of PSC niche cells. Interestingly, *CUX1* is a human homolog of *Drosophila ct* that functions as a haploinsufficient tumor suppressor gene inactivated in acute myeloid leukemia (McNerney *et al.* 2013). This same study showed that haploinsufficiency of *ct* led to hemocyte overproliferation and melanotic tumor formation in *Drosophila* larvae. Thus, Ct has been identified in two studies as being an important hematopoietic factor negatively controlling PSC cell and hemocyte production.

#### longitudinals lacking (lola):

The GMR44C05 line failed to show GFP expression in lymph gland PSC cells isolated from third instar larvae grown under normal conditions (Figure 2I). However, upon wasp challenge, GMR44C05 lymph glands showed a *de novo* reporter expression in PSC cells (Figure 2J). This line contains an enhancer corresponding to *lola* gene DNA. We have previously identified *lola* as a positive regulator of PSC formation based on its RNAi knockdown phenotype (Tokusumi *et al.* 2012). In this study, we further confirmed an importance for the BTB class transcriptional regulator in PSC cells in that gain-of-function *lola* due to *col-Gal4 > UAS lola* expression led to a strong increase in PSC cell number (Figure 6D). Strikingly, this forced expression of Lola, specifically in PSC cells, led to a copious production of lamellocytes in otherwise wild-type lymph glands (Figure 6D). This latter finding suggested that Lola may be inducing a signaling molecule(s), secreted from niche cells, that leads to lamellocyte production and differentiation (Crozatier *et al.* 2004; Krzemień *et al.* 2007; Sinenko *et al.* 2012).

#### hairy (h):

The GMR12H06 line showed GFP expression in MZ cells marked by domeMESO (Figure 3B). This line contains an enhancer corresponding to *h* gene DNA. *h* is best known as a pair rule gene controlling embryonic segmentation. MZ cell-specific knockdown of *h* function by *TepIV-Gal4 > UAS h* RNAi expression resulted in a total loss of the Tep IV-positive prohemocyte population (Figure 6F). *H* is known to function as a transcriptional repressor through its interactions with corepressor proteins like CtBP and Groucho (Abed *et al.* 2011; Poortinga *et al.* 1998). These results implicated *h* as being involved in hemocyte progenitor quiescence by repressing genes that promote the differentiation of hemocytes.

#### Btk family kinase at 29A (Btk29A):

The GMR26D06 line showed GFP expression in a subset of MZ cells marked by domeMESO (Figure 3F). This line contains an enhancer corresponding to the *Btk29A* gene, which encodes a Btk class protein kinase (Hamada-Kawaguchi *et al.* 2014). MZ cell-specific knockdown of *Btk29A* function by *TepIV-Gal4 > UAS Btk29A* RNAi expression resulted in a strong reduction of the TepIV-positive MZ cell population (Figure 6G). These results suggested that Btk29A kinase is expressed in cells of the MZ domain, where it may function in a signaling cascade facilitating the production of prohemocytes.

#### dFOXO:

**Figure 7:**
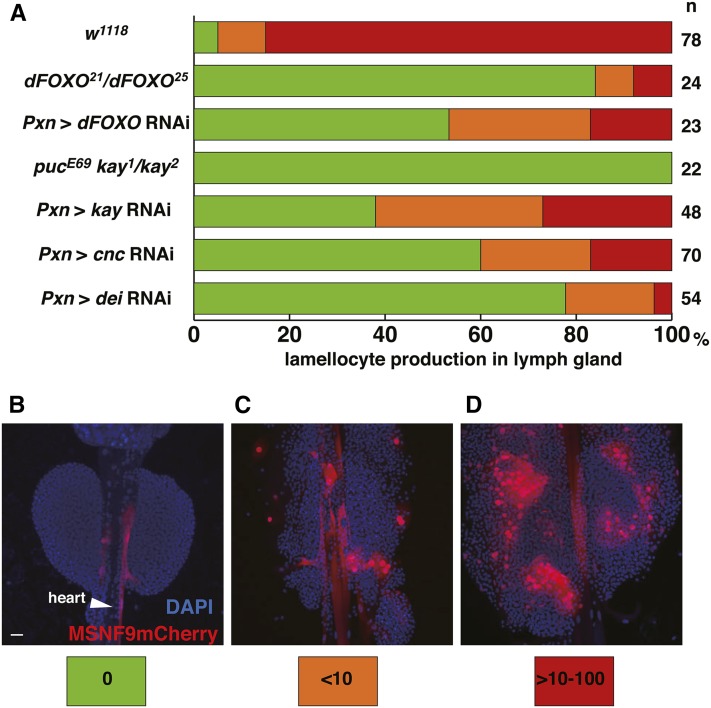
(A) Requirement of the *dFOXO*, *kay*, *cnc*, and *dei* genes for a full cellular immune response to wasp infestation of larvae. (B–D) Quantification of lamellocyte production in lymph glands of the above tested genotypes in response to wasp parasitization. Three values are assigned. (B) Green indicates no (0) lamellocytes observed in assayed lymph glands. (C) Orange indicates a few (1–9) lamellocytes observed in assayed lymph glands. (D) Red indicates a strong induction (>10–100) of lamellocytes in assayed lymph glands. Bar, 20 μm in all images. DAPI, 4’,6-diamidino-2-phenylindole; RNAi, RNA interference.

The GMR60B06 line contains an enhancer from the *dFOXO* gene and failed to express the GFP reporter in lymph glands isolated from control third instar larvae (Figure 5A). However, when animals of this genotype were subjected to wasp challenge, the *dFOXO* enhancer was strongly activated in lamellocytes (Figure 5B). We confirmed the requirement of FOXO function for lamellocyte induction due to wasp infestation (Figure 7A). In addition, we demonstrated that forced expression of FOXO in Pxn^+^ hemocytes via *Pxn-Gal4 > UAS dFOXO* expression resulted in an induction of lamellocytes in lymph glands and hemolymph (Figure 6I and data not shown). Thus, a *dFOXO* enhancer is activated in lamellocytes, this transcriptional regulator is required for a full lamellocyte induction in response to wasp parasitization, and FOXO can dominantly induce the defensive lamellocyte population when expressed in an otherwise wild-type lymph gland.

#### kayak (kay):

The GMR42E11 line contains an enhancer from the *kay* gene that, like the *dFOXO* GMR60B06 line, failed to express GFP in control lymph glands (Figure 5C). In addition, like the *dFOXO* enhancer, the *kay* enhancer was activated in lamellocytes induced upon wasp infestation (Figure 5D). *kay* encodes the *Drosophila* homolog of the mammalian Fos transcription factor, which is a proven proto-oncogene and known target of JNK pathway signaling. Previous studies from our lab have demonstrated that *kay* function is required for the activity of a lamellocyte-specific enhancer of the *misshapen* (*msn*) gene and that *kay* haploinsufficiency reduces *hop^Tum−l^*-induced lamellocyte production (Tokusumi *et al.* 2009b). Other previous studies have shown that forced *kay* expression can dominantly induce lamellocytes (Stofanko *et al.* 2008). In this study, we further demonstrated that *kay* function knockdown by *Pxn-Gal4 > UAS kay* RNAi expression led to a strong reduction in lamellocyte production post-wasp infestation, and that the transheterozygous *kay^1^ puc^E69^/kay^2^* mutant combination led to a complete absence of lamellocyte induction under these wasp challenge conditions (Figure 7A; Ciapponi and Bohmann 2002). Together, these findings demonstrated that a *kay* enhancer is activated in wasp-induced lamellocytes, the dFos transcription factor is required for the activity of the lamellocyte-specific *msn* enhancer, *kay* function is required for lamellocyte induction in response to wasp challenge, and forced gene expression can induce the defensive lamellocyte population in lymph glands.

#### cap-n-collar (cnc):

As noted previously, the GMR36G01 line contains an enhancer from the *cnc* gene, with this enhancer inactive in cells of control lymph gland (Figure 2K). However, upon wasp challenge, this enhancer becomes active in PSC cells and defensive lamellocytes (Figure 2L). The *cnc* gene produces three mRNA isoforms: *cncA*, *cncB*, and *cncC* (McGinnis *et al.* 1998). Interestingly, forced expression of either *cncB* (Figure 6J) or *cncC* isoforms in Pxn-positive hemocytes resulted in a copious production of lamellocytes. In support of the role of one or more *cnc* isoforms in lamellocyte differentiation, *cnc* function knockdown of all isoforms by *Pxn-Gal4 > UAS cnc* RNAi expression resulted in a strong reduction of lamellocyte induction in response to wasp infestation (Figure 7A). Thus, like *dFOXO* and *kay*, *cnc* is a gene possessing an enhancer that is activated in response to wasp challenge to larvae, two mRNA isoforms can dominantly induce the lamellocyte lineage, and gene function is required for a defensive cellular immune response to wasp parasitization.

#### delilah (dei):

The GMR39F12 line contains an enhancer from the *dei* gene and failed to express GFP in any cells of control lymph glands (Figure 5E). But, as seen with the GMR60B06 (*dFOXO* enhancer) and GMR42E11 (*kay* enhancer) strains, the *dei* enhancer became activated in lamellocytes in response to larval wasp challenge (Figure 5F). Additionally, we demonstrated that knockdown of *dei* function led to a strong decrease in lamellocyte production in response to wasp infestation (Figure 7A). *dei* encodes a bHLH-class transcription factor shown to be important for the expression of a βPS integrin subunit required for proper wing formation (Egoz-Matia *et al.* 2011). The same subunit is expressed in lamellocytes, and the Dei transcription factor may likewise be crucial for integrin gene expression and lamellocyte differentiation. Lamellocytes also express the αPS4 integrin, but the function of these integrin subunits in these blood cells has yet to be investigated (Stofanko *et al.* 2010).

#### E2F transcription factor 1 (E2f1):

E2F1 is a transcription factor involved in cell cycle control and numerous studies have shown that this protein interacts with the DP protein, with the E2F1/DP heterodimeric transcription factor complex positively regulating many genes required for initiation of S phase of the cell cycle. We have previously demonstrated that this regulator controls PSC cell number through its direct or indirect activation of the *dMyc* cell proliferation gene (Tokusumi *et al.* 2015). In the current study, it was confirmed that *E2f1* loss-of-function via *col-Gal4 > UAS-E2f1* RNAi expression resulted in a strong decrease in PSC cell number, while *E2f1* gain-of-function via *col-Gal4 > UAS E2f1* expression culminated in a significant increase in niche cells (Table 1). Thus, a clear function for this gene has been established in the control of PSC cell number.

**Figure 8:**
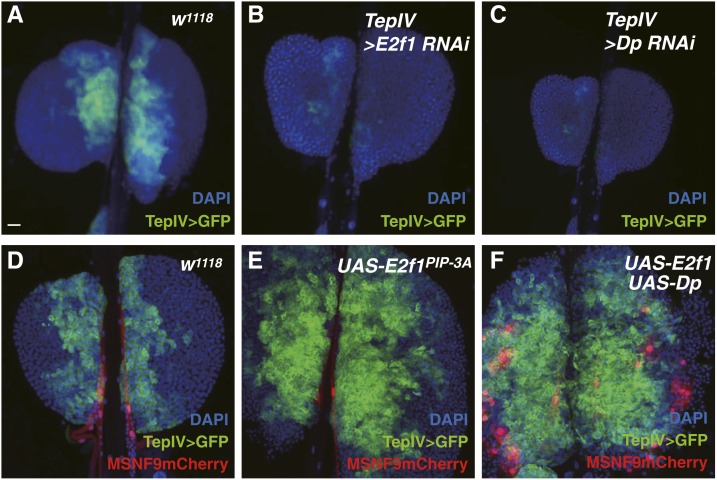
Lymph gland phenotypes in *E2f1* and *Dp* gain- and loss-of-function lymph glands from third instar larvae. (A and D) The *TepIVGal4 > UASmCD8GFP* combination is used to identify a normal population of progenitors (green) in wild-type lymph glands. *MSNF9mCherry* marks lamellocytes (red, none detected). (B and C) Abrogation of *E2f1* or *Dp* function by RNAi knockdown leads to a complete loss of stem cell-like prohemocytes. (E) Gain-of-function *E2f1* leads to a massive overproduction of hematopoietic progenitors (green). (F) Gain-of-function *E2f1* and *Dp* results in a massive expansion of the prohemocyte population (green) and the high-level inducement of lamellocytes (red). Bar, 20 μm in all images. DAPI, 4’,6-diamidino-2-phenylindole; GFP, green fluorescent protein; RNAi, RNA interference.

It was of interest that we also observed GFP expression in MZ cells in the GMR47F05 line (Figure 3A), as this line contains an enhancer corresponding to *E2f1* gene DNA. To test for a possible function of the E2F1 regulator in these blood cell progenitors, we again conducted gene gain- and loss-of-function analyses. As for cell-specific loss-of-function mediated by RNAi expression, the abrogation of either *E2f1* or *Dp* functions resulted in a complete loss of the TepIV-positive prohemocyte pool (Figure 8, B and C). Conversely, forced expression of a *UAS-E2f1* cDNA in MZ cells resulted in a robust overproduction of hematopoietic progenitors (Figure 8E). Even more dramatic was the result obtained with the coexpression of *E2f1* and *Dp* under the control of the *TepIVGal4* driver: a massive expansion of pluripotent prohemocytes and the copious induction of lamellocytes as well (Figure 8F). Thus, this expression combination increased both the hematopoietic progenitor population and induced specialized hemocytes involved in innate immune responses. One explanation is that the level of the activating E2F1/DP transcription factor complex was elevated relative to the level of the inhibitory RBF protein. Together, these findings suggested the importance of the E2F1 and DP cell cycle regulators for hematopoietic progenitor production and maintenance, and the positive control of lamellocyte induction.

### Conclusions

We have undertaken an extensive screen for enhancer-Gal4 lines that are active in cells of the larval hematopoietic organ and in differentiated hemocytes. A total of 190 enhancers associated with 87 genes were shown to be expressed in cells of the larval lymph gland and/or blood cells of the larval hemolymph. Although we believe that many Gal4 lines are an accurate indicator of enhancer-associated gene expression, Gal4 expression patterns may sometimes differ from RNA patterns detected by *in situ* hybridization or protein expression by antibody immunostaining. Thus, they may not fully reflect actual gene expression. Therefore, in future studies, it may be wise to confirm enhancer activity with additional gene expression analyses such as RNA and/or protein detection in lymph glands for a gene of interest.

Most of the 87 genes were analyzed for hematopoietic phenotypes through gain- and/or loss-of-function studies. Except for a few genes, we mainly used RNAi lines in gene loss-of-function analyses. It is noted that this approach may occasionally be prone to off-target effects, so a detailed analysis of the function of a gene in hematopoietic cells would benefit from the phenotypic analysis of gene mutants. Nonetheless, we have found multiple examples of the cellular location of enhancer activity being consistent with the loss-of-function phenotype generated through the use of a gene-specific RNAi reagent. Select highlights of these expression and phenotype analyses are elaborated as follows.

Numerous enhancer-Gal4 lines have been identified that are expressed exclusively in PSC cells, MZ cells, or mature hemocytes of the CZ or hemolymph. On occasion, one may try to utilize a certain supposed tissue-specific enhancer for gene gain- or loss-of-function studies, but due to unfortunate activity of the said enhancer in additional cells, the driver-expression combination may result in lethality prior to a developmental time point of interest. With the identification of multiple cell-specific enhancer-Gal4 lines in the current study, the experimental repertoire of hematopoietic system genetic tools has been substantially increased.

Several unique enhancer-Gal4 lines were discovered in this screen. One is the GMR40F10 strain, which contains an enhancer from the *bbx* gene (Figure 4L). This enhancer-Gal4 combination is active in blood cells of the hemolymph but not in lymph gland CZ hemocytes. Using this driver and a cell death-inducing gene such as *UAS-hid*, one can selectively ablate hemolymph blood cells while leaving the lymph gland population unharmed. The effect of eliminating this functionally distinct blood cell population and tissue (Leitão and Sucena 2015; Markus *et al.* 2009) can be assayed for in larvae grown under normal *vs.* physiologically challenged conditions.

The current study allowed us to identify the *lola* gene as a potent regulator of lamellocyte induction and differentiation. Previous work from our lab had shown that *lola* functioned as a positive regulator of PSC formation (Tokusumi *et al.* 2012), and the current analysis demonstrated that a *lola* enhancer in the GMR44C05 strain was activated in niche cells upon wasp parasitization of larvae (Figure 2J). Surprisingly, forced expression of the Lola BTB class transcription factor in PSC cells led to a copious production of lamellocytes in otherwise normal lymph glands (Figure 6D). It has been shown that Spitz is a cytokine factor secreted from oxidatively challenged PSC niche cells, where it functions to induce lamellocyte production from CZ cells (Sinenko *et al.* 2012). It is possible that forced Lola expression leads to Spitz and/or another cytokine factor that signals from the PSC niche to promote CZ cells to undergo lamellocyte differentiation. Making this connection would provide informative mechanistic information as to how *lola* is controlling the induction of this defensive cell type.

Lamellocyte production serves as a cellular innate immune response to wasp infestation of *Drosophila* larvae. The current study identified, or further supported, the requirement of four genes for a competent defensive response to wasp challenge. These include the *dFOXO*, *kay*, *cnc*, and *dei* genes. Coupled with our findings on *lola*, these results indicate that lamellocyte production in response to wasp parasitization is a genetically complex larval response. The cellular origin of lamellocytes has been investigated, with some results implicating plasmatocytes as the source (Honti *et al.* 2010; Stofanko *et al.* 2010). Other findings point to the crystal cell lineage as the origin (Ferguson and Martinez-Agosto 2014; Krzemien *et al.* 2010). Yet another study argues for a subepidermal population of sessile blood cells as being the source of induced lamellocytes (Markus *et al.* 2009). Thus, one explanation of our discovery of multiple genes required for lamellocyte induction in response to wasp challenge is that different gene products may be working in distinct cell types to alter the plasticity of cells of origin to achieve the lamellocyte fate. Another possibility is that transcriptional regulators may be functioning in a combinatorial manner in the production and differentiation of the lamellocyte population.

## Supplementary Material

Supplemental material is available online at www.g3journal.org/lookup/suppl/doi:10.1534/g3.116.034439/-/DC1.
