# Supplementary material for: Screening and Analysis of Janelia FlyLight Project Enhancer-Gal4 Strains Identifies Multiple Gene Enhancers Active During Hematopoiesis in Normal and Wasp-Challenged Drosophila Larvae
Source: G3 (Bethesda). 2016 Dec 1;7(2):437–48. doi: 10.1534/g3.116.034439 (PMC5295592; doi:10.1534/g3.116.034439)
Supplement: Supplementary file 1 [file 437FigureS1.pdf]

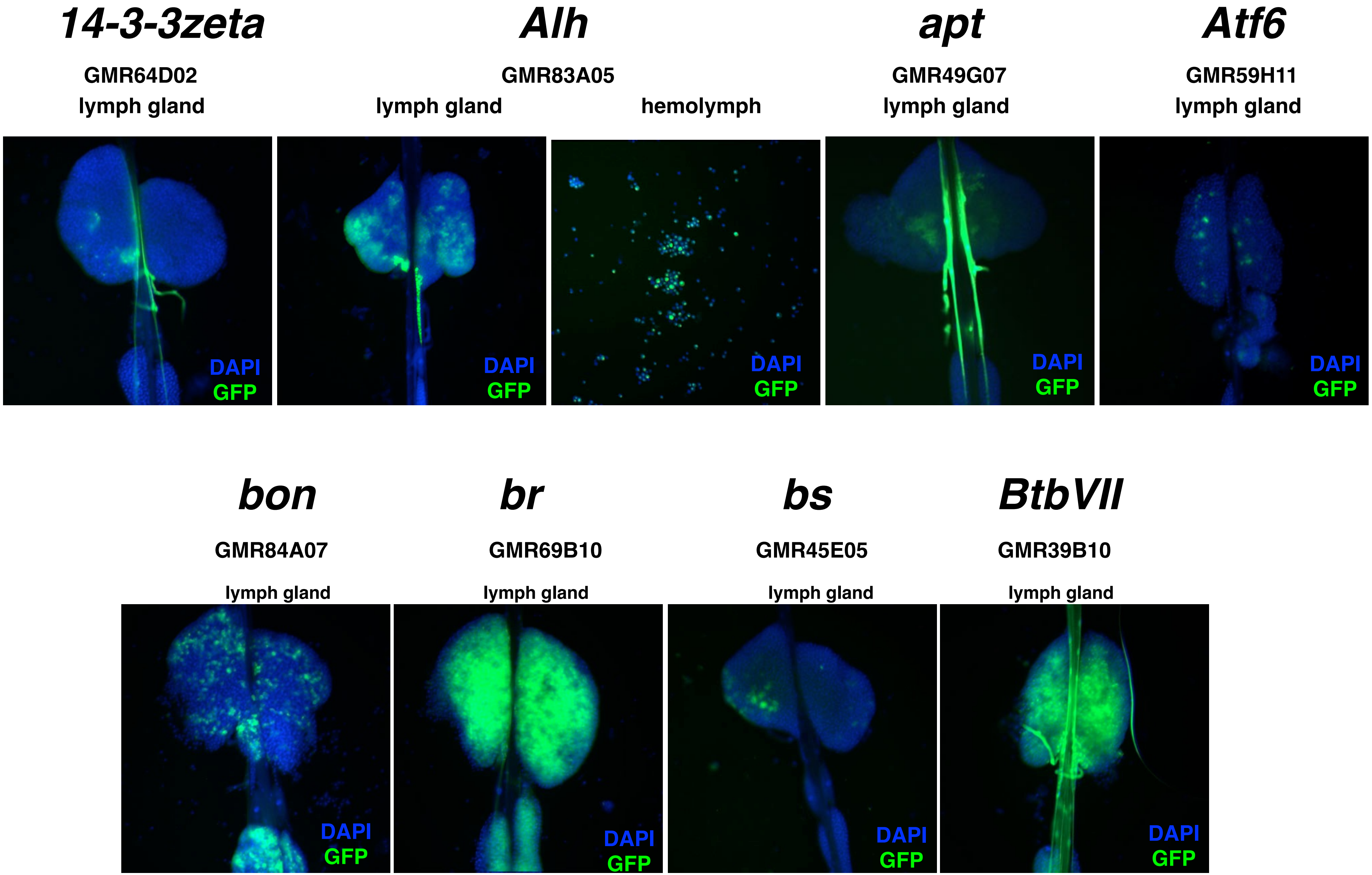
14-3-3zeta
GMR64D02
lymph gland
DAPI
GFP
Alh
GMR83A05
lymph gland
DAPI
GFP
hemolymph
DAPI
GFP
apt
GMR49G07
lymph gland
DAPI
GFP
Atf6
GMR59H11
lymph gland
DAPI
GFP
bon
GMR84A07
lymph gland
DAPI
GFP
br
GMR69B10
lymph gland
DAPI
GFP
bs
GMR45E05
lymph gland
DAPI
GFP
BtbVII
GMR39B10
lymph gland
DAPI
GFP

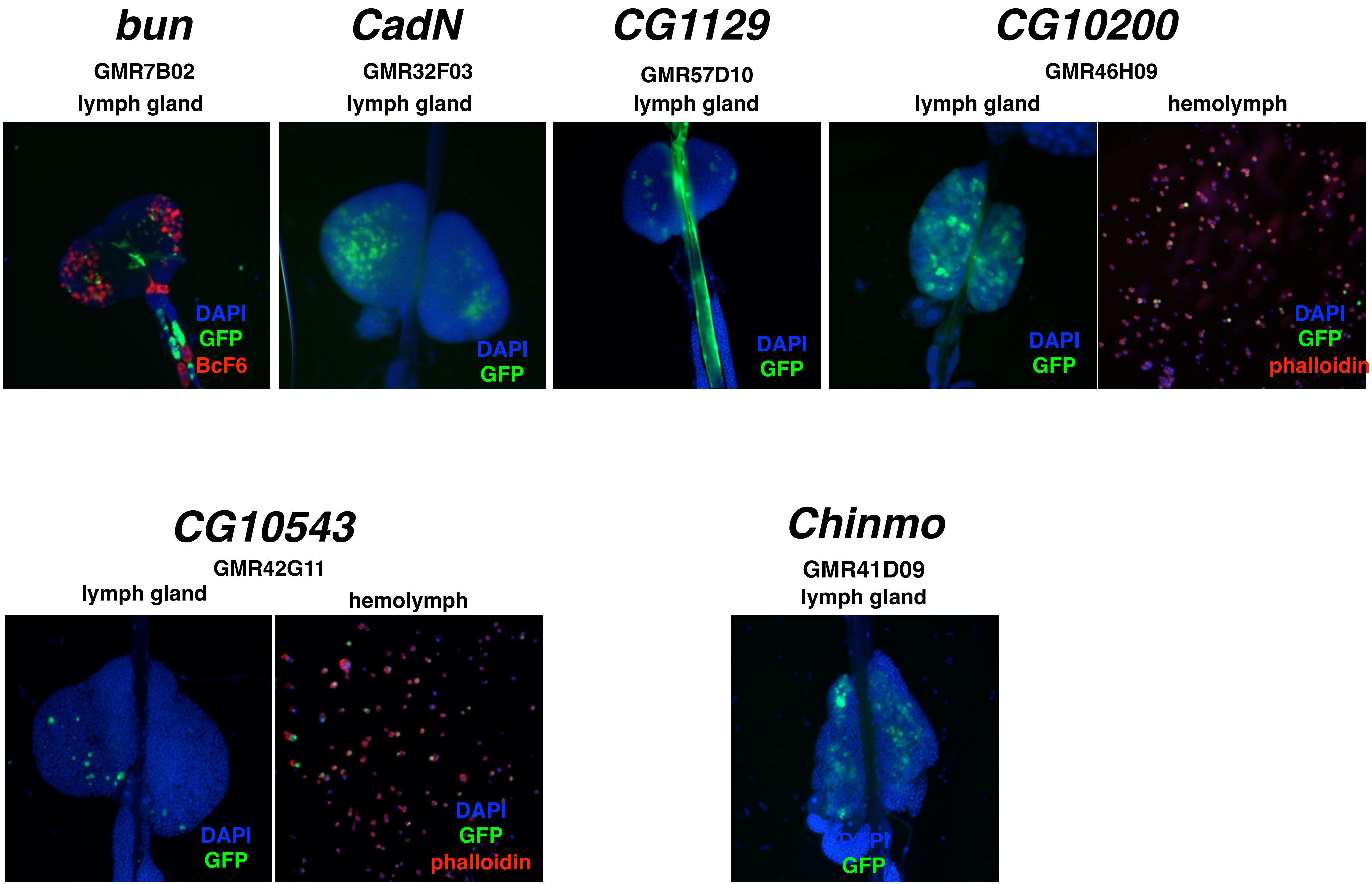
bun
GMR7B02
lymph gland
DAPI
GFP
BcF6
CadN
GMR32F03
lymph gland
DAPI
GFP
CG1129
GMR57D10
lymph gland
DAPI
GFP
CG10200
GMR46H09
lymph gland
DAPI
GFP
hemolymph
DAPI
GFP
phalloidin
CG10543
GMR42G11
lymph gland
DAPI
GFP
hemolymph
DAPI
GFP
phalloidin
Chinmo
GMR41D09
lymph gland
GFP

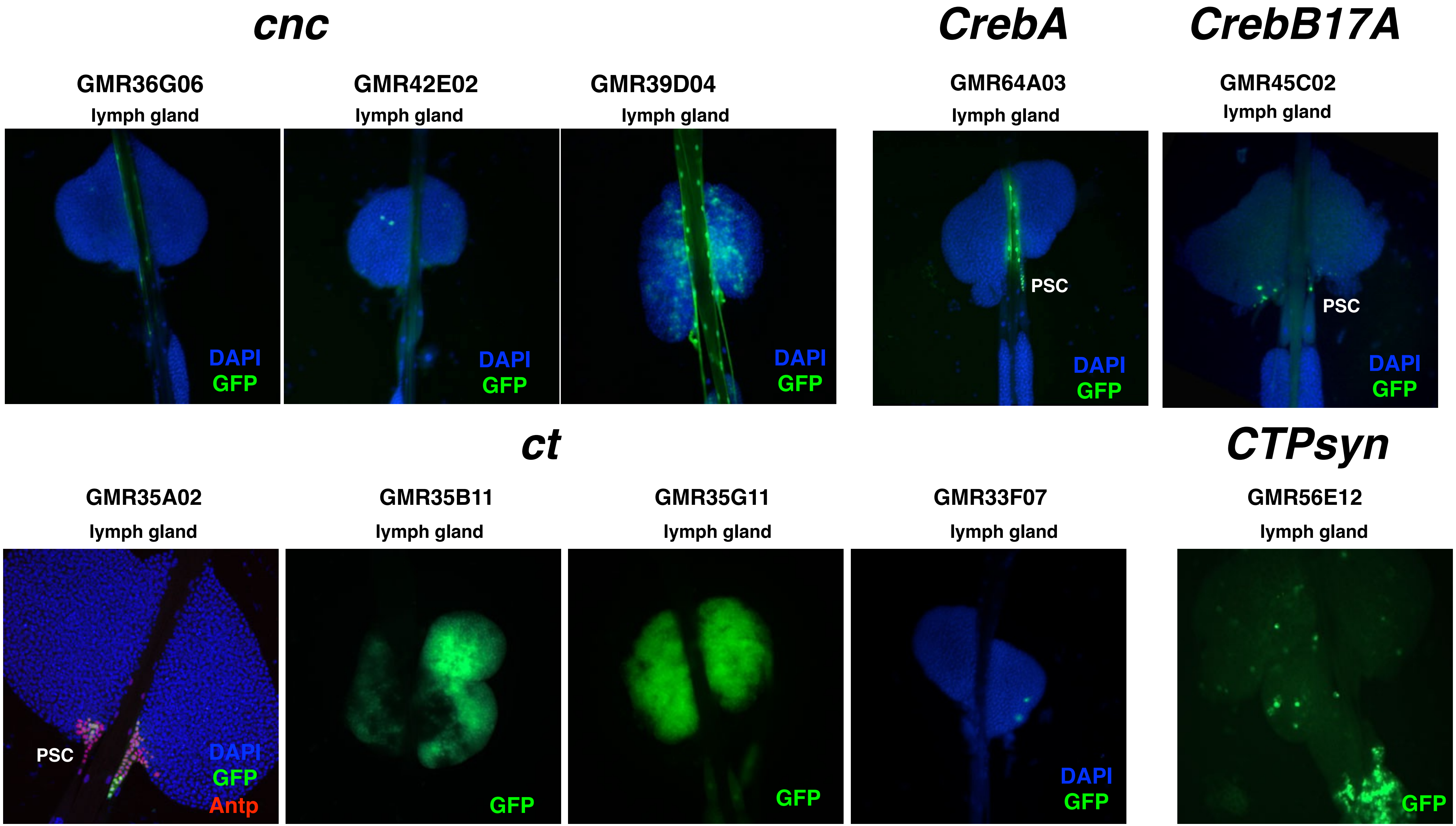

cnc
CrebA
CrebB17A
GMR36G06
lymph gland
DAPI
GFP
GMR42E02
lymph gland
DAPI
GFP
GMR39D04
lymph gland
DAPI
GFP
GMR64A03
lymph gland
PSC
DAPI
GFP
GMR45C02
lymph gland
PSC
DAPI
GFP
ct
CTPsyn
GMR35A02
lymph gland
PSC
DAPI
GFP
Antp
GMR35B11
lymph gland
GFP
GMR35G11
lymph gland
GFP
GMR33F07
lymph gland
DAPI
GFP
GMR56E12
lymph gland
GFP

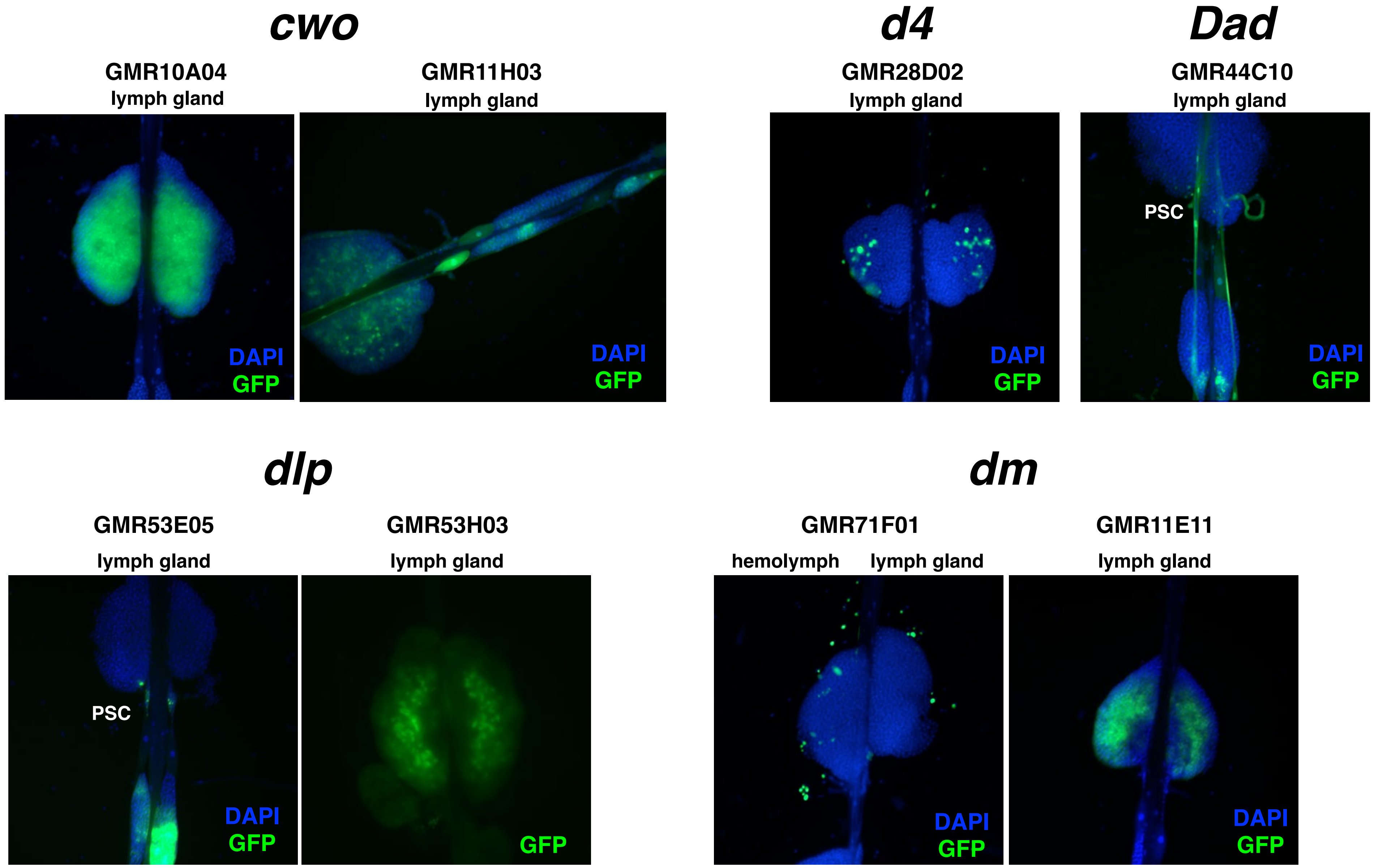
cwo
GMR10A04
lymph gland
DAPI
GFP
GMR11H03
lymph gland
DAPI
GFP
d4
GMR28D02
lymph gland
DAPI
GFP
Dad
GMR44C10
lymph gland
PSC
DAPI
GFP
dlp
GMR53E05
lymph gland
PSC
DAPI
GFP
GMR53H03
lymph gland
GFP
dm
GMR71F01
hemolymph
lymph gland
DAPI
GFP
GMR11E11
lymph gland
DAPI
GFP

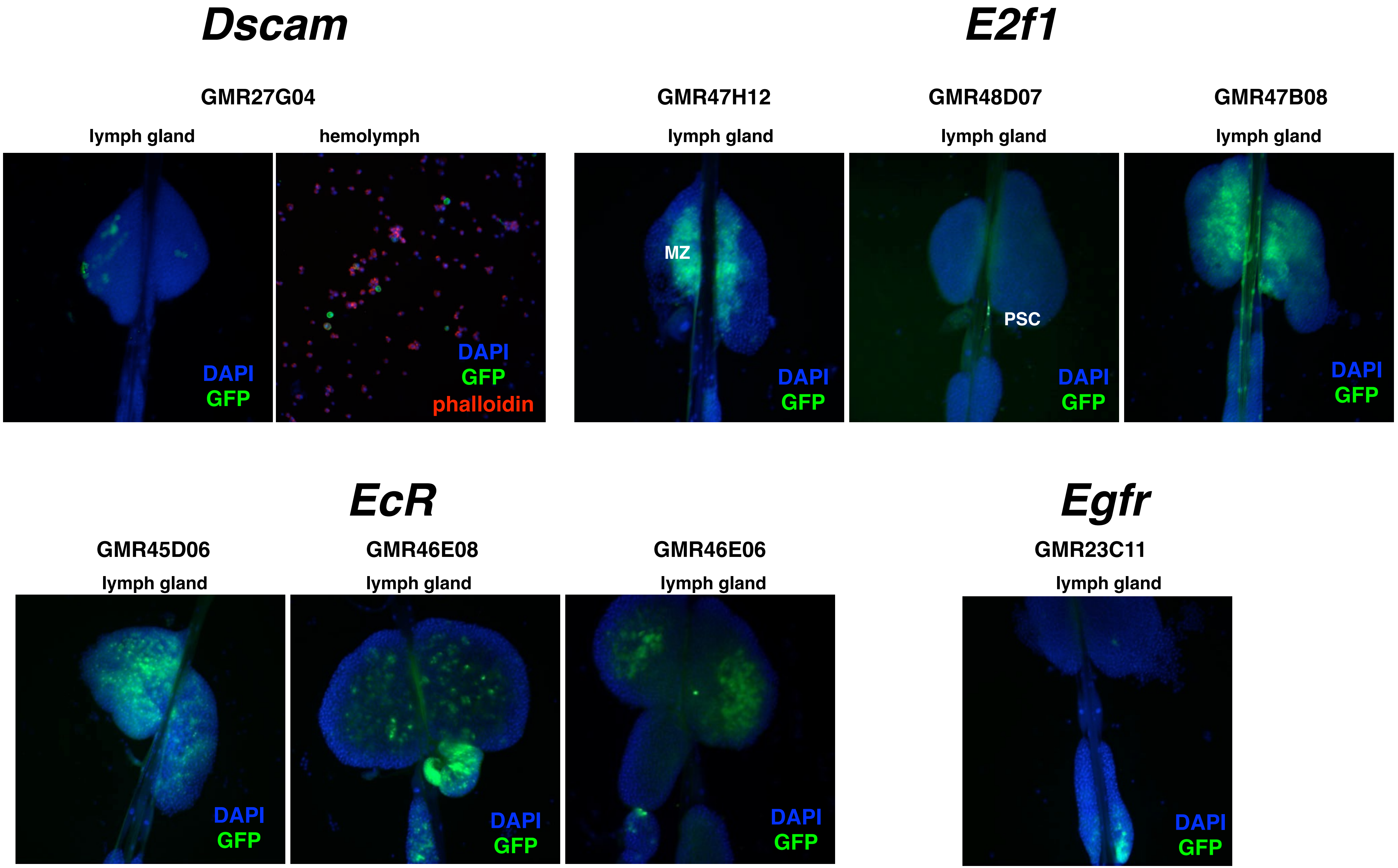

Dscam
GMR27G04
lymph gland
DAPI
GFP
hemolymph
DAPI
GFP
phalloidin
E2f1
GMR47H12
lymph gland
MZ
DAPI
GFP
GMR48D07
lymph gland
PSC
DAPI
GFP
GMR47B08
lymph gland
DAPI
GFP
EcR
GMR45D06
lymph gland
DAPI
GFP
GMR46E08
lymph gland
DAPI
GFP
GMR46E06
lymph gland
DAPI
GFP
Egfr
GMR23C11
lymph gland
DAPI
GFP

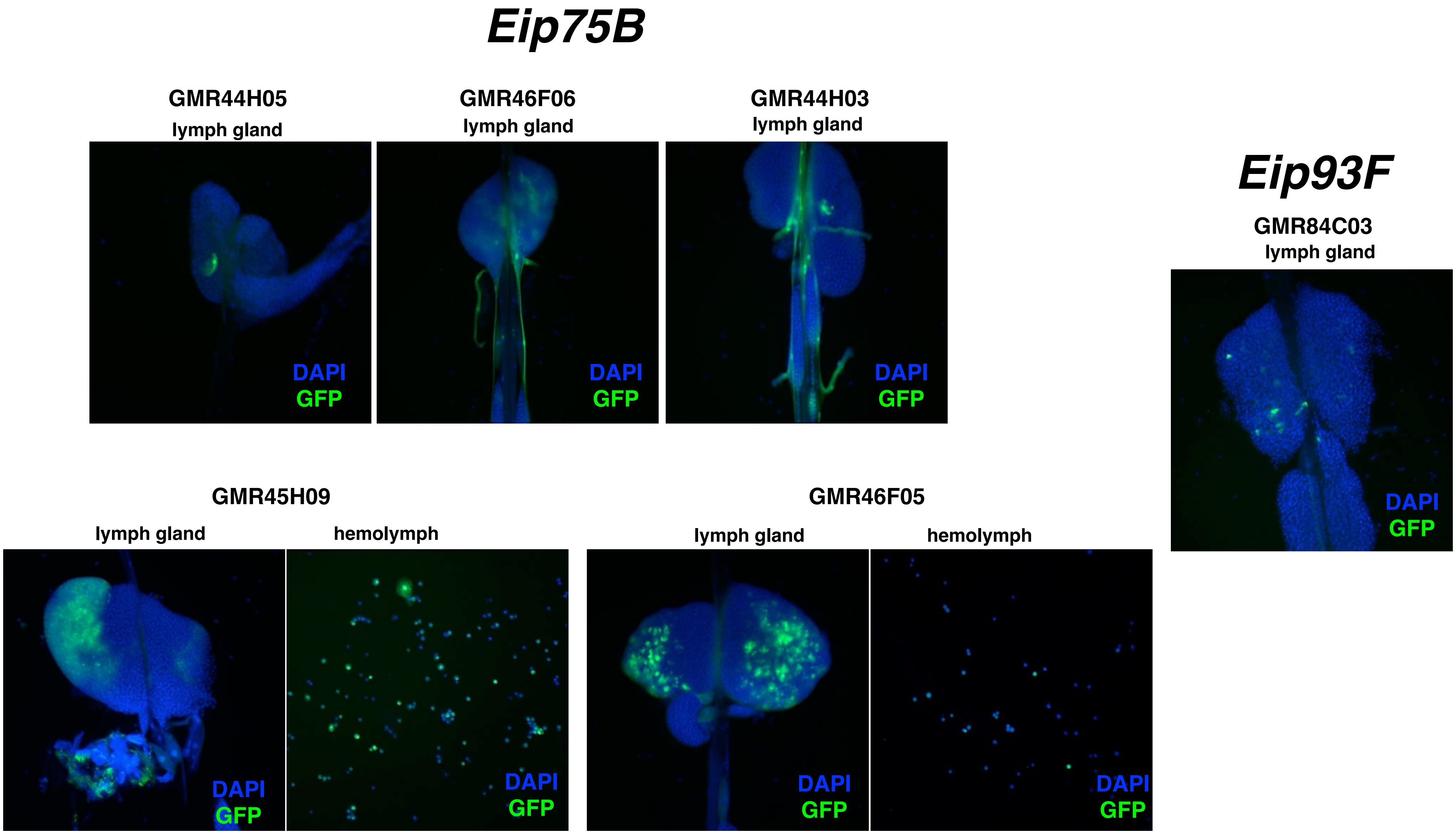
Eip75B
GMR44H05
lymph gland
DAPI
GFP
GMR46F06
lymph gland
DAPI
GFP
GMR44H03
lymph gland
DAPI
GFP
Eip93F
GMR84C03
lymph gland
DAPI
GFP
GMR45H09
lymph gland
DAPI
GFP
hemolymph
DAPI
GFP
GMR46F05
lymph gland
DAPI
GFP
hemolymph
DAPI
GFP

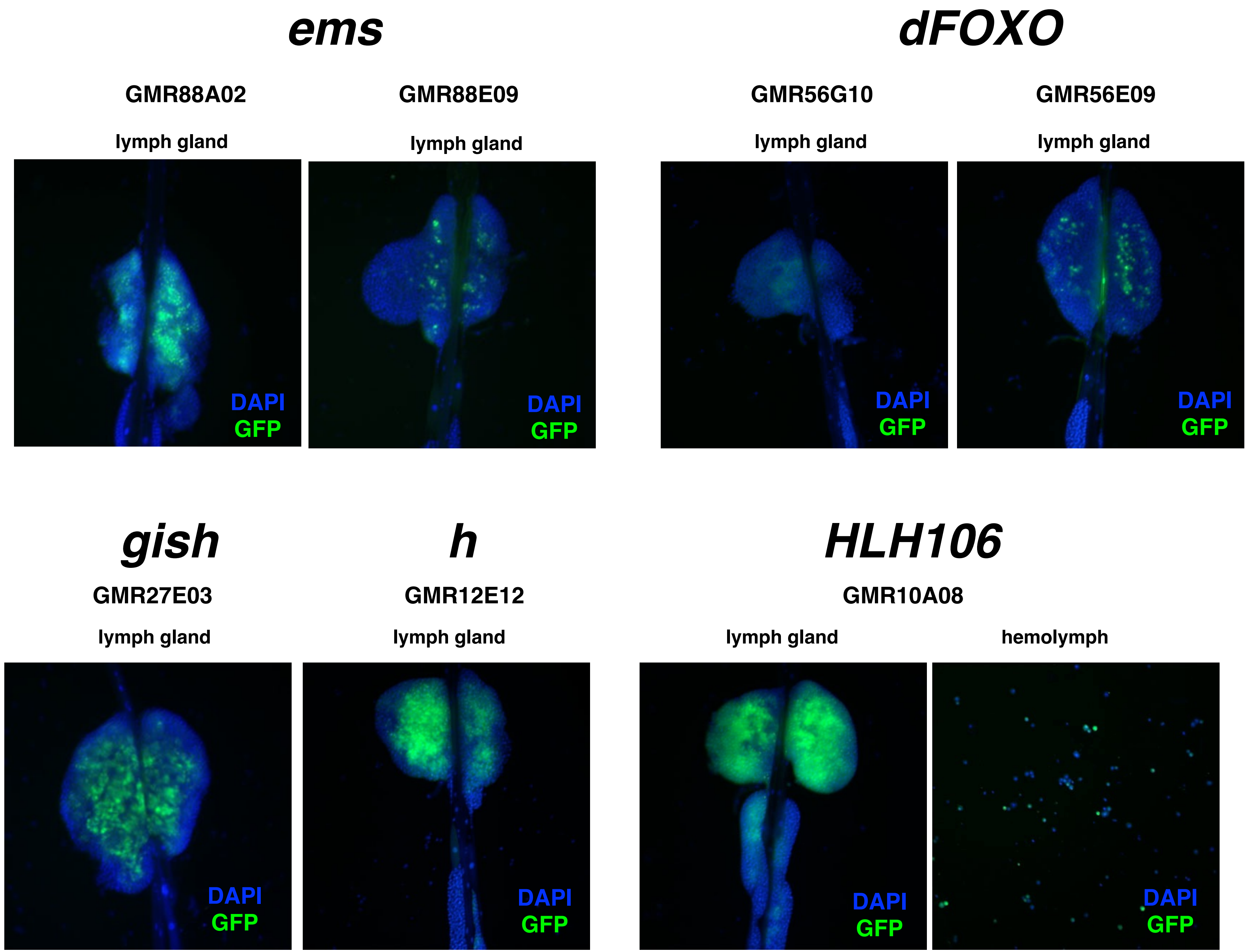

ems
dFOXO
GMR88A02
GMR88E09
GMR56G10
GMR56E09
lymph gland
lymph gland
lymph gland
lymph gland
DAPI
GFP
DAPI
GFP
DAPI
GFP
DAPI
GFP
gish
h
HLH106
GMR27E03
GMR12E12
GMR10A08
lymph gland
lymph gland
lymph gland
hemolymph
DAPI
GFP
DAPI
GFP
DAPI
GFP
DAPI
GFP

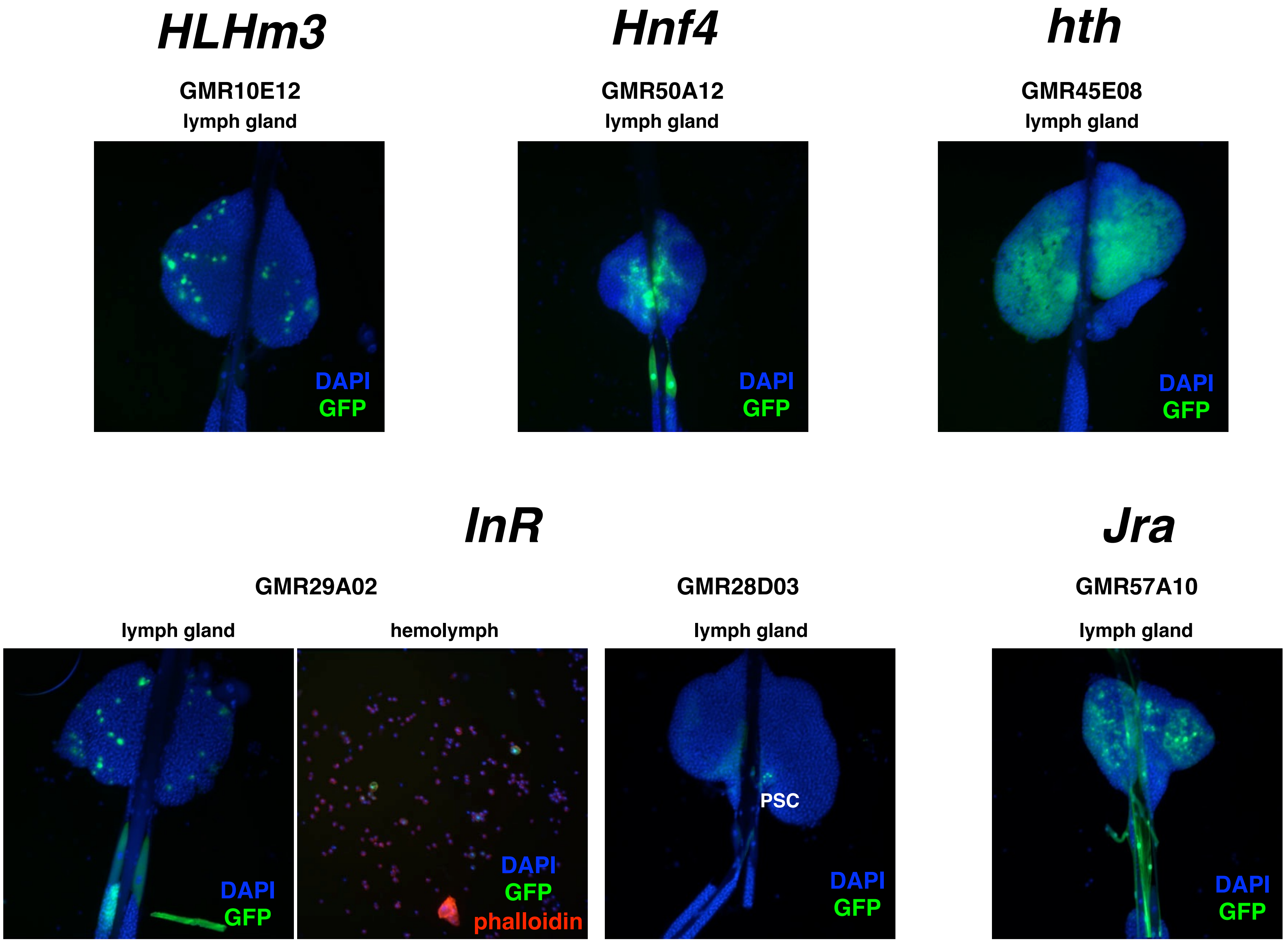

HLHm3
GMR10E12
lymph gland
DAPI
GFP
Hnf4
GMR50A12
lymph gland
DAPI
GFP
hth
GMR45E08
lymph gland
DAPI
GFP
InR
GMR29A02
lymph gland
DAPI
GFP
hemolymph
DAPI
GFP
phalloidin
GMR28D03
lymph gland
PSC
DAPI
GFP
Jra
GMR57A10
lymph gland
DAPI
GFP

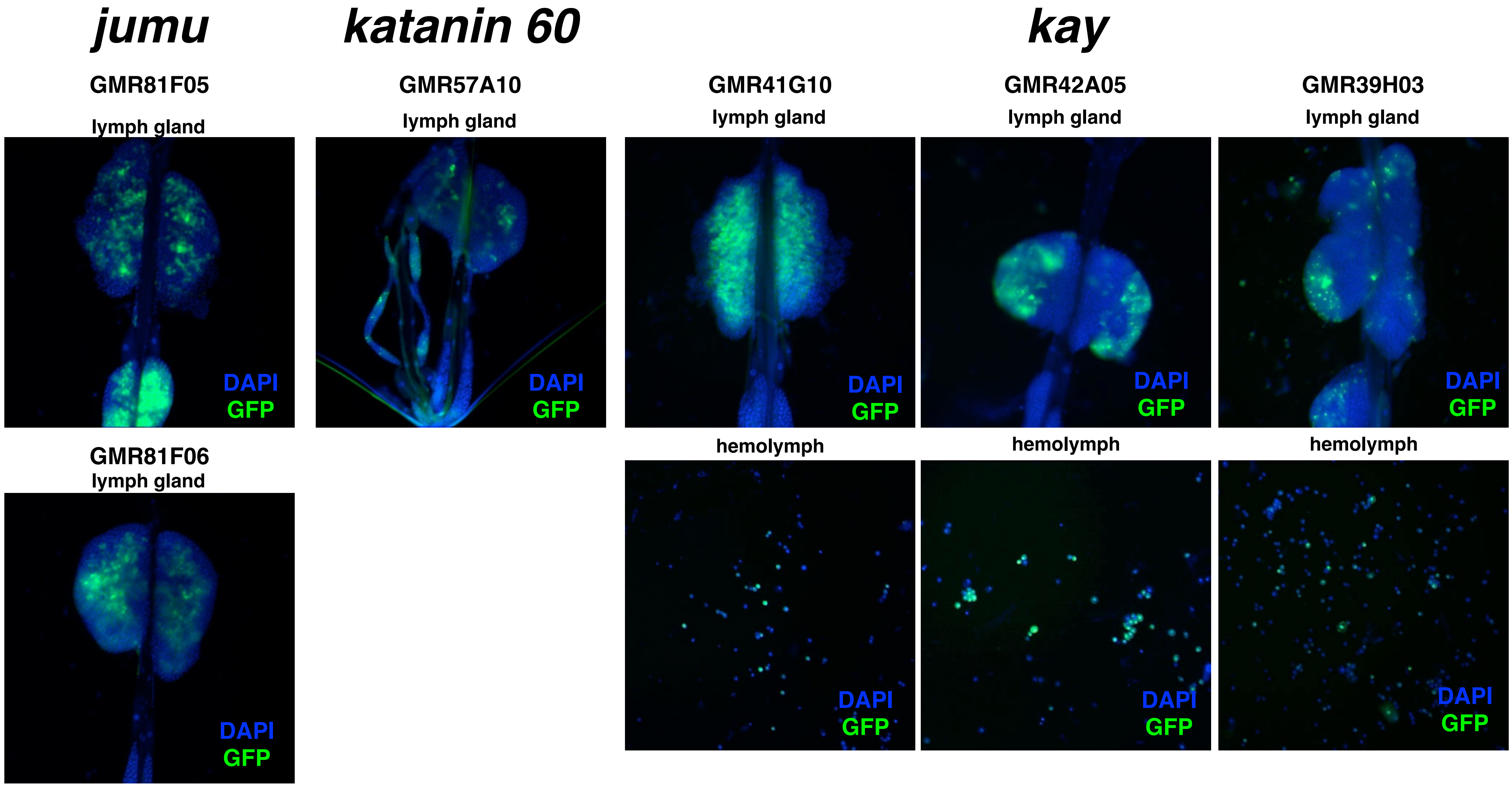
jumu
katanin 60
kay
GMR81F05
lymph gland
GMR57A10
lymph gland
GMR41G10
lymph gland
GMR42A05
lymph gland
GMR39H03
lymph gland
GMR81F06
lymph gland
hemolymph
hemolymph
hemolymph
DAPI
GFP

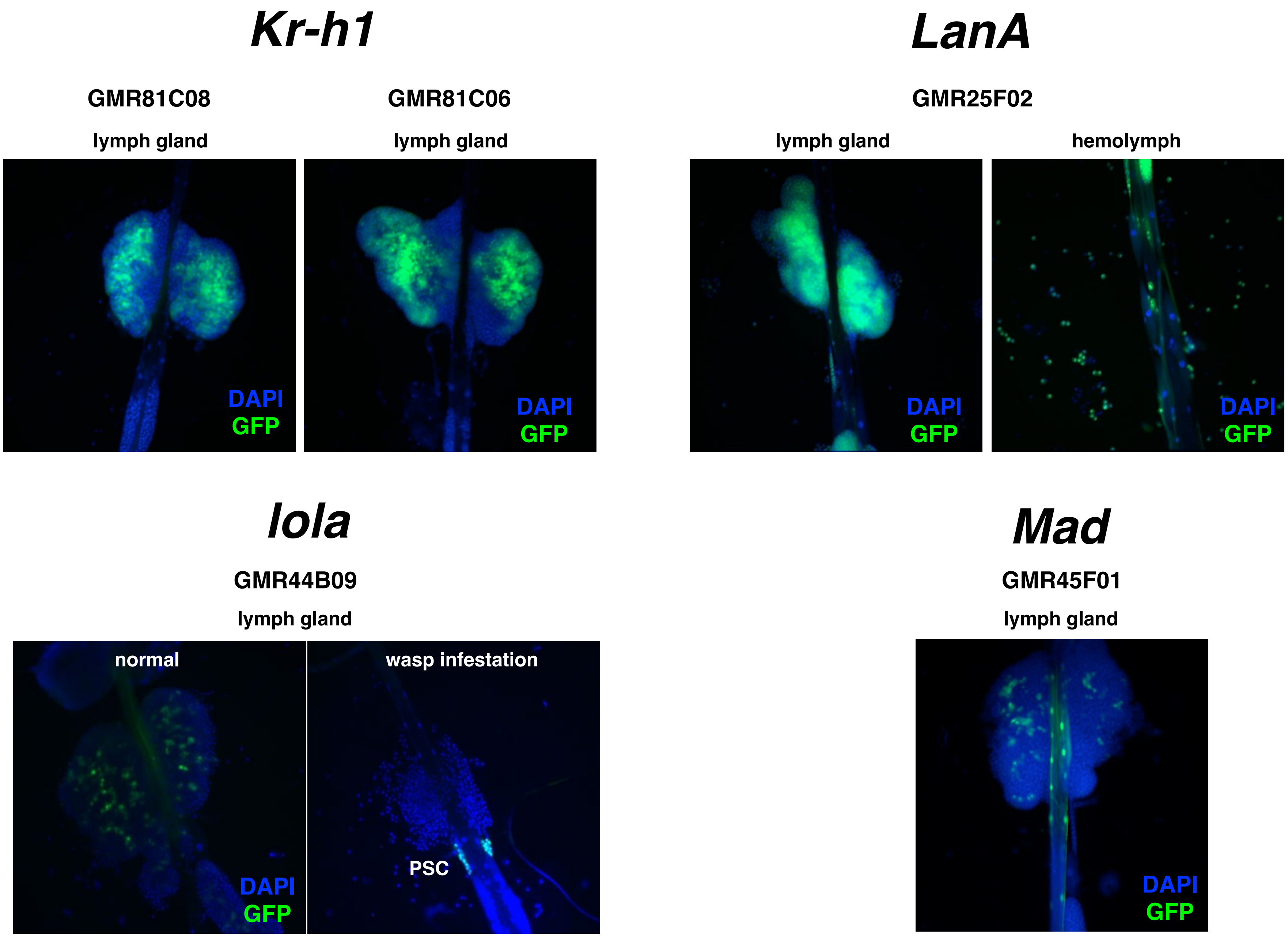
Kr-h1
GMR81C08
lymph gland
DAPI
GFP
GMR81C06
lymph gland
DAPI
GFP
LanA
GMR25F02
lymph gland
DAPI
GFP
hemolymph
DAPI
GFP
lola
GMR44B09
lymph gland
normal
DAPI
GFP
wasp infestation
PSC
Mad
GMR45F01
lymph gland
DAPI
GFP

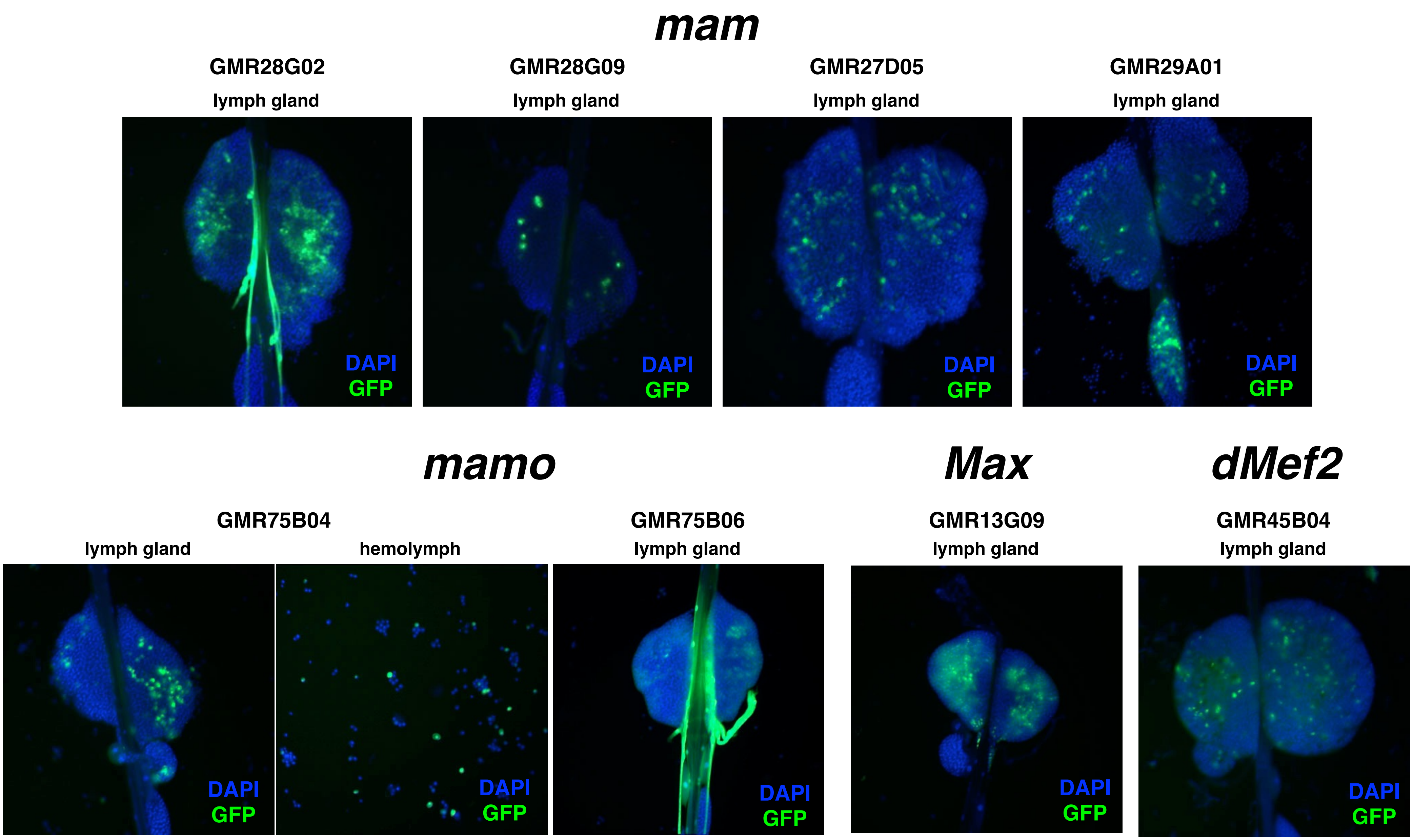
mam
GMR28G02
lymph gland
GMR28G09
lymph gland
GMR27D05
lymph gland
GMR29A01
lymph gland
DAPI
GFP
mamo
GMR75B04
lymph gland
hemolymph
GMR75B06
lymph gland
Max
GMR13G09
lymph gland
dMef2
GMR45B04
lymph gland

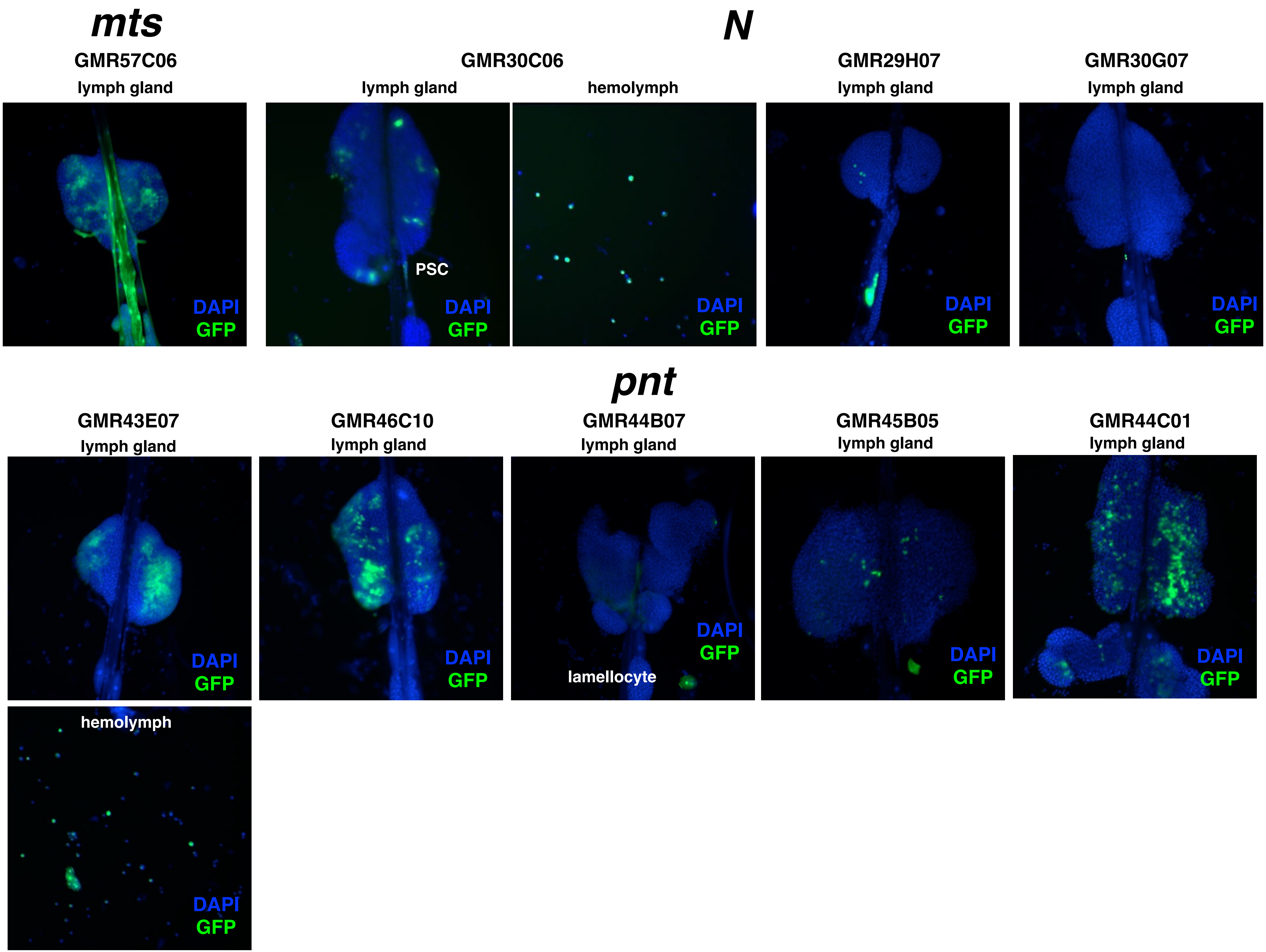
mts
N
GMR57C06
lymph gland
GMR30C06
lymph gland
hemolymph
PSC
GMR29H07
lymph gland
GMR30G07
lymph gland
pnt
GMR43E07
lymph gland
hemolymph
GMR46C10
lymph gland
GMR44B07
lymph gland
lamellocyte
GMR45B05
lymph gland
GMR44C01
lymph gland
DAPI
GFP

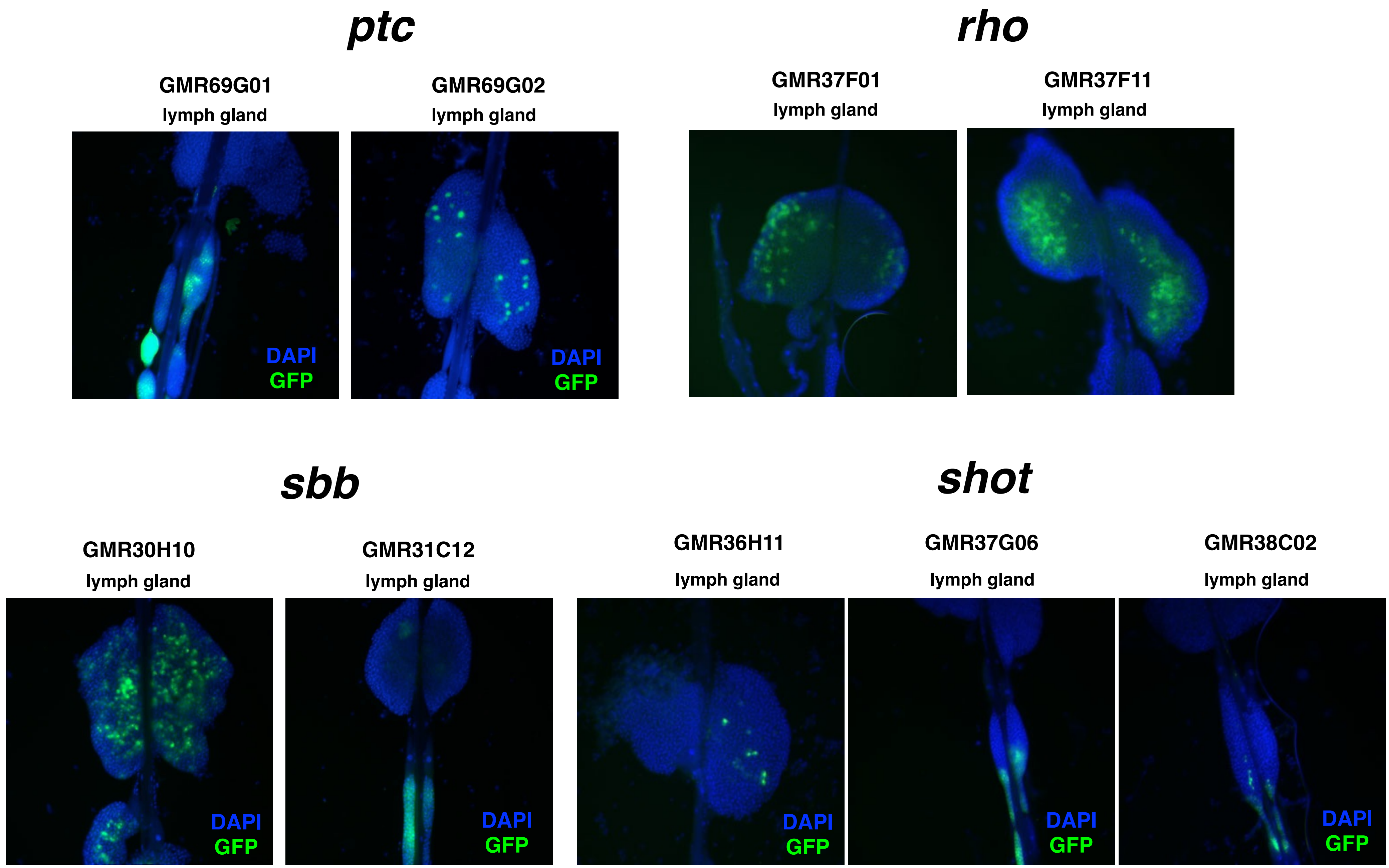
ptc
GMR69G01
lymph gland
DAPI
GFP
GMR69G02
lymph gland
DAPI
GFP
rho
GMR37F01
lymph gland
GMR37F11
lymph gland
sbb
GMR30H10
lymph gland
DAPI
GFP
GMR31C12
lymph gland
DAPI
GFP
shot
GMR36H11
lymph gland
DAPI
GFP
GMR37G06
lymph gland
DAPI
GFP
GMR38C02
lymph gland
DAPI
GFP

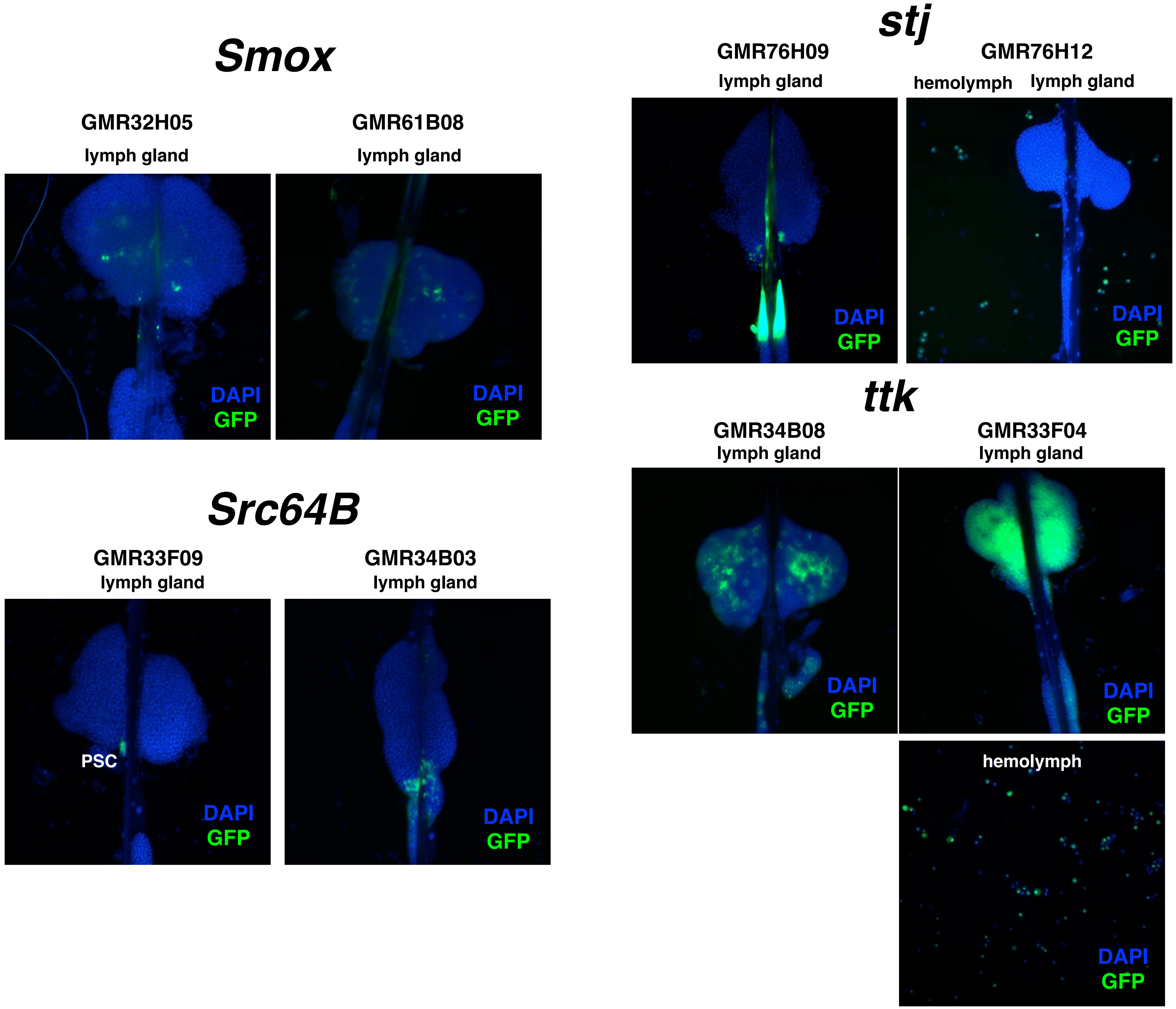
Smox
GMR32H05
lymph gland
DAPI
GFP
GMR61B08
lymph gland
DAPI
GFP
Src64B
GMR33F09
lymph gland
PSC
DAPI
GFP
GMR34B03
lymph gland
DAPI
GFP
stj
GMR76H09
lymph gland
DAPI
GFP
GMR76H12
hemolymph
lymph gland
DAPI
GFP
ttk
GMR34B08
lymph gland
DAPI
GFP
GMR33F04
lymph gland
DAPI
GFP
hemolymph
DAPI
GFP

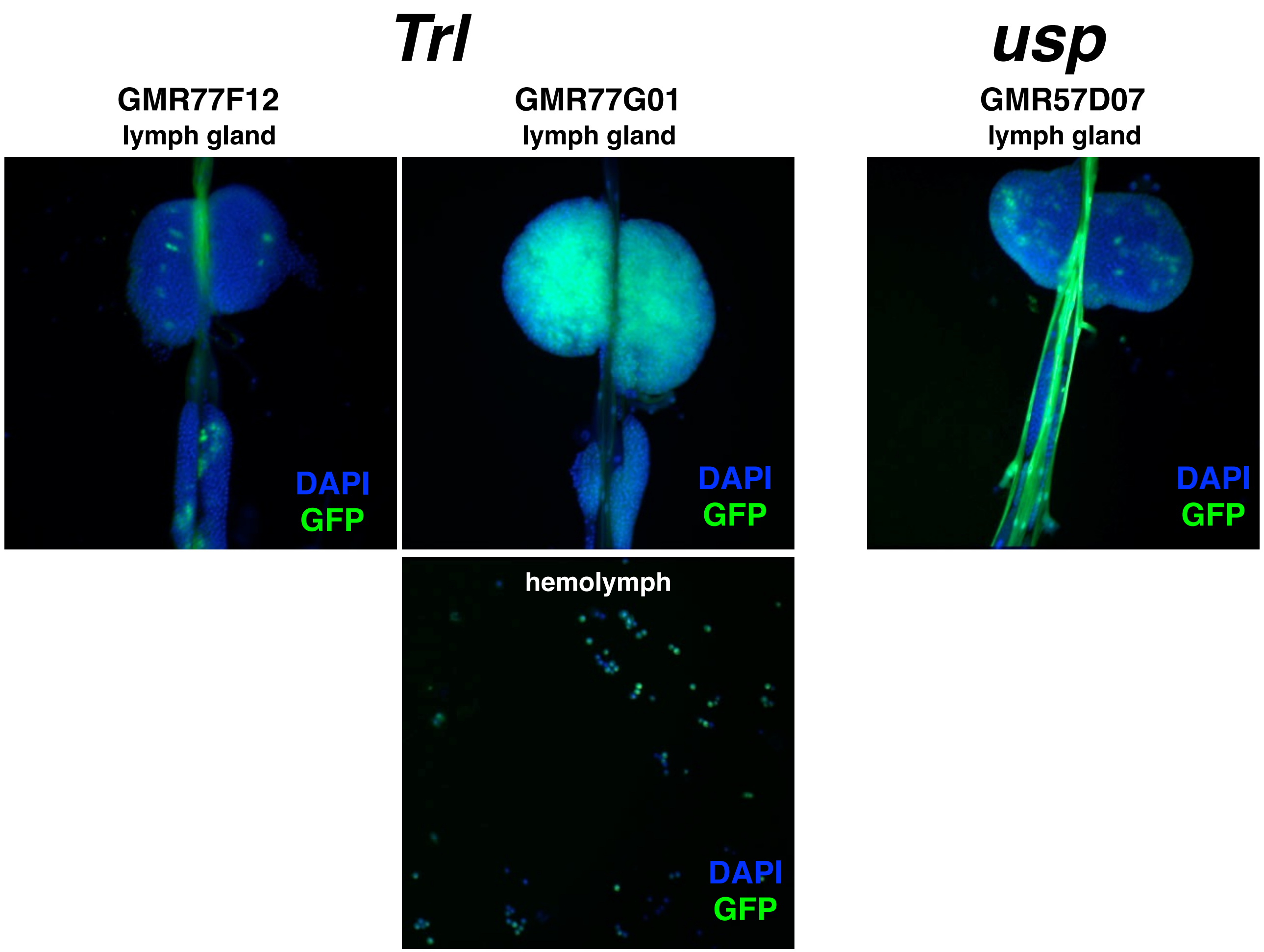
Trl
usp
GMR77F12
lymph gland
GMR77G01
lymph gland
GMR57D07
lymph gland
DAPI
GFP
DAPI
GFP
DAPI
GFP
hemolymph
DAPI
GFP
