# Supplementary material for: Screening and Analysis of Janelia FlyLight Project Enhancer-Gal4 Strains Identifies Multiple Gene Enhancers Active During Hematopoiesis in Normal and Wasp-Challenged Drosophila Larvae
Source: G3 (Bethesda). 2016 Dec 1;7(2):437–48. doi: 10.1534/g3.116.034439 (PMC5295592; doi:10.1534/g3.116.034439)
Supplement: Supplementary file 2 [file 437TableS1.docx]

Table S1. List of all FlyLight Project enhancer-Gal4 lines assayed in this study. (.xlsx, 517 KB)

<http://www.g3journal.org/lookup/suppl/doi:10.1534/g3.116.034439/-/DC1/TableS1.xlsx>
