# Supplementary material for: Screening and Analysis of Janelia FlyLight Project Enhancer-Gal4 Strains Identifies Multiple Gene Enhancers Active During Hematopoiesis in Normal and Wasp-Challenged Drosophila Larvae
Source: G3 (Bethesda). 2016 Dec 1;7(2):437–48. doi: 10.1534/g3.116.034439 (PMC5295592; doi:10.1534/g3.116.034439)
Supplement: Supplementary file 4 [file 437TableS2.docx]

Table S2. Summary of the results of enhancer-Gal4 line screening as to hematopoietic tissue and cell expression pattern of the GFP reporter protein. (.xlsx, 503 KB)

[http://www.g3journal.org/lookup/suppl/doi:10.1534/g3.116.034439/-/DC1/TableS2.xlsx](http://www.g3journal.org/lookup/suppl/doi:10.1534/g3.116.034439/-/DC1/TableS1.xlsx)
