# Supplementary material for: Screening and Analysis of Janelia FlyLight Project Enhancer-Gal4 Strains Identifies Multiple Gene Enhancers Active During Hematopoiesis in Normal and Wasp-Challenged Drosophila Larvae
Source: G3 (Bethesda). 2016 Dec 1;7(2):437–48. doi: 10.1534/g3.116.034439 (PMC5295592; doi:10.1534/g3.116.034439)
Supplement: Supplementary file 6 [file 437TableS3.docx]

Table S3. List of the various *UAS-RNAi* and *UAS-cDNA* lines used in this study in the phenotypic analyses of hematopoietic tissue or blood cell-expressed genes. (.xlsx, 498 KB)

<http://www.g3journal.org/lookup/suppl/doi:10.1534/g3.116.034439/-/DC1/TableS3.xlsx>
